# Impact of sex on the efficacy of immune checkpoint inhibitors in kidney and urothelial cancers: a systematic review and meta-analysis

**DOI:** 10.1007/s00345-023-04412-0

**Published:** 2023-05-20

**Authors:** Takafumi Yanagisawa, Tatsushi Kawada, Fahad Quhal, Kensuke Bekku, Ekaterina Laukhtina, Pawel Rajwa, Markus von Deimling, Muhammad Majdoub, Marcin Chlosta, Benjamin Pradere, Keiichiro Mori, Takahiro Kimura, Manuela Schmidinger, Pierre I. Karakiewicz, Shahrokh F. Shariat

**Affiliations:** 1 Department of Urology, Comprehensive Cancer Center, Medical University of Vienna, Wahringer Gurtel 43 18-20, 1090 Vienna, Austria; 2 Department of Urology, The Jikei University School of Medicine, Tokyo, Japan; 3 Department of Urology, Dentistry and Pharmaceutical Sciences, Okayama University Graduate School of Medicine, Okayama, Japan; 4 Department of Urology, King Fahad Specialist Hospital, Dammam, Saudi Arabia; 5 Institute for Urology and Reproductive Health, Sechenov University, Moscow, Russia; 6 Department of Urology, Medical University of Silesia, Zabrze, Poland; 7 Department of Urology, University Medical Center Hamburg-Eppendorf, Hamburg, Germany; 8 Department of Urology, Hillel Yaffe Medical Center, Hadera, Israel; 9 Clinic of Urology and Urological Oncology, Jagiellonian University, Krakow, Poland; 10 Department of Urology, La Croix Du Sud Hospital, Quint Fonsegrives, France; 11 Cancer Prognostics and Health Outcomes Unit, Division of Urology, University of Montreal Health Center, Montreal, Canada; 12 Division of Urology, Department of Special Surgery, The University of Jordan, Amman, Jordan; 13 Department of Urology, University of Texas Southwestern Medical Center, Dallas, TX USA; 14 Department of Urology, Second Faculty of Medicine, Charles University, Prague, Czech Republic; 15 Department of Urology, Weill Cornell Medical College, New York, NY USA; 16 Karl Landsteiner Institute of Urology and Andrology, Vienna, Austria

**Keywords:** Sex, Gender, Immune checkpoint inhibitors, Renal cell carcinoma, Urothelial carcinoma, Metastatic, Advanced, Adjuvant

## Abstract

**Purpose:**

To analyze and summarize the efficacy of immune checkpoint inhibitor (ICI) alone or in combination therapy for renal cell carcinoma (RCC) and urothelial carcinoma (UC) stratified by sex.

**Methods:**

Three databases were queried in October 2022 for randomized controlled trials (RCTs) analyzing RCC and UC patients treated with ICIs. We analyzed the association between sex and the efficacy of ICIs in RCC and UC patients across several clinical settings. The outcomes of interest were overall survival (OS) and progression-free survival for the metastatic setting and disease-free survival (DFS) for the adjuvant setting.

**Results:**

Overall, 16 RCTs were included for meta-analyses and network meta-analyses. In the first-line treatment of metastatic RCC (mRCC) and UC (mUC) patients, ICI-based combination therapies significantly improved OS compared to the current standard of care, regardless of sex. Adjuvant ICI monotherapy reduced the risk of disease recurrence in female patients with locally advanced RCC (pooled hazard ratio [HR]: 0.71, 95% confidence interval [CI] 0.55–0.93) but not in male patients, and, conversely, in male patients with muscle-invasive UC (pooled HR: 0.80, 95%CI 0.68–0.94) but not in female patients. Treatment ranking analyses in the first-line treatment of mRCC and mUC showed different results between sexes. Of note, regarding adjuvant treatment for RCC, pembrolizumab (99%) had the highest likelihood of improved DFS in males, whereas atezolizumab (84%) in females.

**Conclusions:**

OS benefit of first-line ICI-based combination therapy was seen in mRCC and mUC patients regardless of sex. Sex-based recommendations for ICI-based regimens according to the clinical setting may help guide clinical decision-making.

**Supplementary Information:**

The online version contains supplementary material available at 10.1007/s00345-023-04412-0.

## Introduction

The inclusion of immune checkpoint inhibitors (ICIs) has changed the treatment landscape of metastatic renal cell (mRCC) and urothelial (mUC) carcinoma [1, 2]. Earlier use of these therapies such as in the adjuvant therapy for locally advanced RCC and UC has shown promise in randomized controlled trials (RCTs) [3, 4].

Considering the patients’ sex is one of the first steps towards personalized medicine [5–7]. For example, female sex is established as a prognosticator of worse survival in patients with muscle-invasive bladder UC [7]. In mRCC, similarly, sex-related discrepancies in the distribution of metastases exist [8]. These differences between men and woman suggest that biological, genetic, and social differences between sexes play an important role in the biology and natural history (i.e., response to therapy) of the underlying disease.

Indeed, immunity and immune response varies among sexes [9, 10], as demonstrated in several cancers, such as glioblastoma or non-small cell lung cancer [11, 12]. A pan-cancer meta-analysis including melanoma and non-small cell lung cancer showed that the overall survival (OS) benefit from ICI was significantly worse in female patients than in male patients [13]. However, these data suffer from disease heterogeneity. Specifically, pooled data on sex-specific differences in the efficacy of ICI focusing on urologic cancers is scarce [14]. Therefore, we conducted this systematic review, meta-analysis, and network meta-analysis (NMA) to comprehensively assess the sex-specific differential efficacy of the ICI monotherapy or ICI-based combination therapies on survival outcomes of urologic cancers in both the metastatic and adjuvant settings. Based on different biology, we separately analyzed RCC and UC patients.

## Methods

The protocol has been registered in the International Prospective Register of Systematic Reviews database (PROSPERO: CRD42022368243).

### Search strategy

This systematic review, meta-analysis, and NMA was conducted based on the guidelines of the Preferred Reporting Items for Meta-Analyses of Observational Studies in Epidemiology Statement (Supplementary Table 1)[15]. A literature search on PubMed^®^, Web of Science™, and Scopus^®^ databases was performed in October 2022 to identify studies investigating the oncologic outcomes of ICI monotherapy or ICI-based combination therapies for RCC or UC. The detailed search strategy is described in Supplementary Appendix. Abstracts presented at recent major conferences were reviewed to include unpublished RCTs and trials’ updates. The outcome measurements of interest were OS and progression-free survival (PFS) for the metastatic setting and disease-free survival (DFS) for the adjuvant setting. Two investigators independently performed the initial screening based on the titles and abstracts to identify eligible studies. Disagreements were resolved by consensus with co-authors.

### Inclusion and exclusion criteria

Studies were included if they investigated RCC and UC patients (Patients) and compared the efficacy of the ICI monotherapy or ICI-based combination therapy (Interventions) with the efficacy of standard of care (SOC) at the time of study enrollment (Comparisons) to assess their differential effects on OS and/or PFS between sexes (Outcome) in an RCT (Study design). Studies lacking original patient data, reviews, letters, editorial comments, replies from authors, case reports, and articles not written in English were excluded. References of all papers included were scanned for additional studies of interest.

### Data extraction

The following data were independently extracted by two authors; studies and the first author’s name, publication year, inclusion criteria, agents, number of patients stratified by sex, follow-up periods, International Metastatic RCC Database Consortium (IMDC) classification and objective response rates (ORRs) for mRCC patients. Subsequently, the hazard ratios (HRs) and 95% confidence intervals (CIs) for OS and/or PFS, or DFS were retrieved. The IMmotion151 trial, which compared the efficacy of atezolizumab + bevacizumab versus sunitinib in previously untreated mRCC, did not provide data on differential oncologic outcomes stratified by sex [16]. In addition, the IMvigor211 trial, which compared the efficacy of atezolizumab versus chemotherapy in mUC, also did not provide data on relevant oncologic outcomes; therefore, these two RCTs were excluded [17].

### Risk of bias assessment

Assessment of study quality and risk of bias was conducted according to the Cochrane Handbook for Systematic Reviews of Interventions risk-of-bias tool (RoB version 2) (Supplementary Fig. 1)[18]. The risk-of-bias assessment of each study was performed independently by two authors.

### Statistical analyses

For meta-analysis, forest plots with HRs were used to analyze the association between ICI therapy and oncologic outcomes. PFS was defined as the time from treatment initiation to radiological progression evaluated by investigator-assessed Response Evaluation Criteria In Solid Tumors version 1.1 (RECIST v1.1), clinical progression, or death. DFS was defined as the time from randomization to the first documented local or distant recurrence or death, whichever occurred first. A fixed-effect model was used for calculations of HRs [19]. Heterogeneity among the outcomes of included studies in this meta-analysis was assessed using Cochrane’s Q test. When significant heterogeneity (p-value of < 0.05 in Cochrane’s Q test) was observed, we attempted to investigate the cause of heterogeneity [20]. Funnel plots were created to evaluate the publication bias using Review Manager 5.3 Software (RevMan; The Cochrane Collaboration, Oxford, UK, Supplementary Fig. 2).

For network meta-analysis, we performed a network meta-analysis using random-effect models with a frequentist approach for direct and indirect treatment comparisons with regard to OS, PFS, and DFS [21, 22]. In the assessment of oncologic outcomes, contrast-based analyses were applied with estimated differences in the log HR and the standard error calculated from the published HR and CI [23]. The relative effects were presented as HRs and 95% CI [21]. We also estimated the relative ranking of the different regimens for OS, PFS, and DFS using the surface under the cumulative ranking (SUCRA) [21].

All analyses were conducted using R version 4.0.5 (R Foundation for Statistical Computing, Vienna, Austria), and the statistical significance level was set at P < 0.05.

## Results

### Study selection and characteristics

Our initial search identified 4,654 records. After removing duplicates, 2,533 records remained for screening titles and abstracts (Supplementary Fig. 3). After screening, a full-text review of 48 articles was performed. According to the inclusion criteria, we finally identified 16 RCTs (19 publications) eligible for meta-analyses or NMAs [3, 4, 24–40]. Of the 16 RCTs, nine included RCC patients [3, 24–34] and seven included UC patients [4, 35–40]. The demographics of each included study are shown in Supplementary Table 2 and 3. There were 2,038 female patients out of 7,615 patients (27%) in the RCC studies and 1,181 out of 5,000 patients (24%) in the UC studies.

### RCC

#### Study selection and characteristics

The study demographics and oncologic outcomes of included studies are shown in Supplementary Table 2. Among nine studies included, five studies comprising 4,206 patients, assessed ICI-based combination therapy in mRCC as 1st-line treatment [24, 25, 27–29, 31, 33], one study investigated ICI monotherapy in mRCC in the 2nd-or 3rd line of therapy for disease progression after tyrosine kinase inhibitors (TKIs) [26], and three studies comprising 2,588 patients evaluated ICI monotherapy or combination therapy in locally advanced RCC as adjuvant therapy [3, 30, 32, 34]. Control arms of all studies of 1st line ICI-based combination therapy were sunitinib, however, the control arm of the CheckMate025 trial for 2nd- or 3rd-line therapy of mRCC was everolimus.

#### Meta-analysis

The results of our meta-analyses and NMAs are summarized in Supplementary Table 4.

##### Efficacy of ICI-based combination therapy for mRCC

*OS*: Systemic therapy with ICI significantly reduced the risk of overall mortality in male (pooled HR: 0.75, 95%CI: 0.67–0.84) and female patients (pooled HR: 0.67, 95%CI: 0.56–0.80) compared to TKI or mTOR inhibitors (Supplementary Fig. 1). There was no significant difference between male and female patients in terms of the OS benefit with ICI (p = 0.3).

**Fig. 1:**
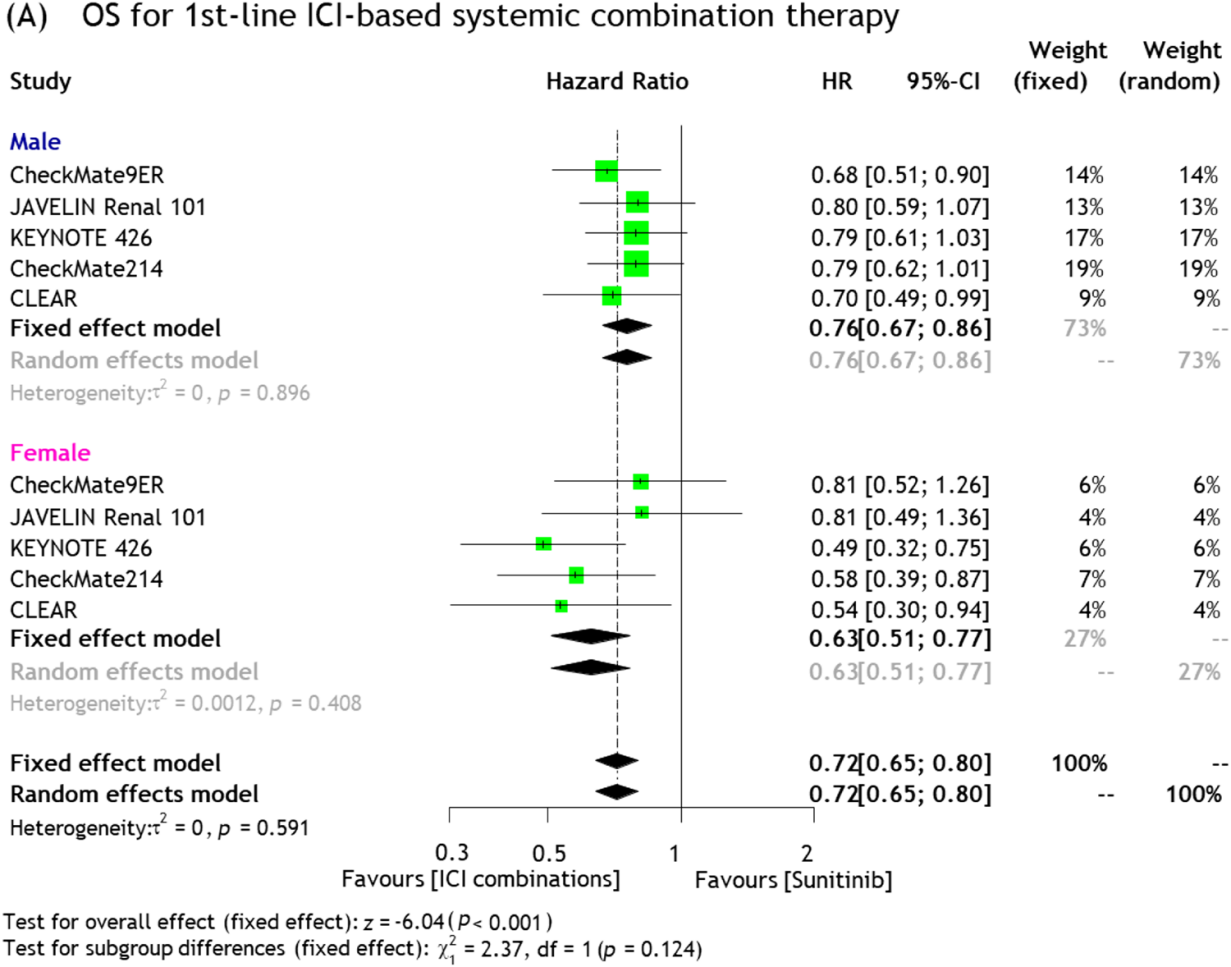

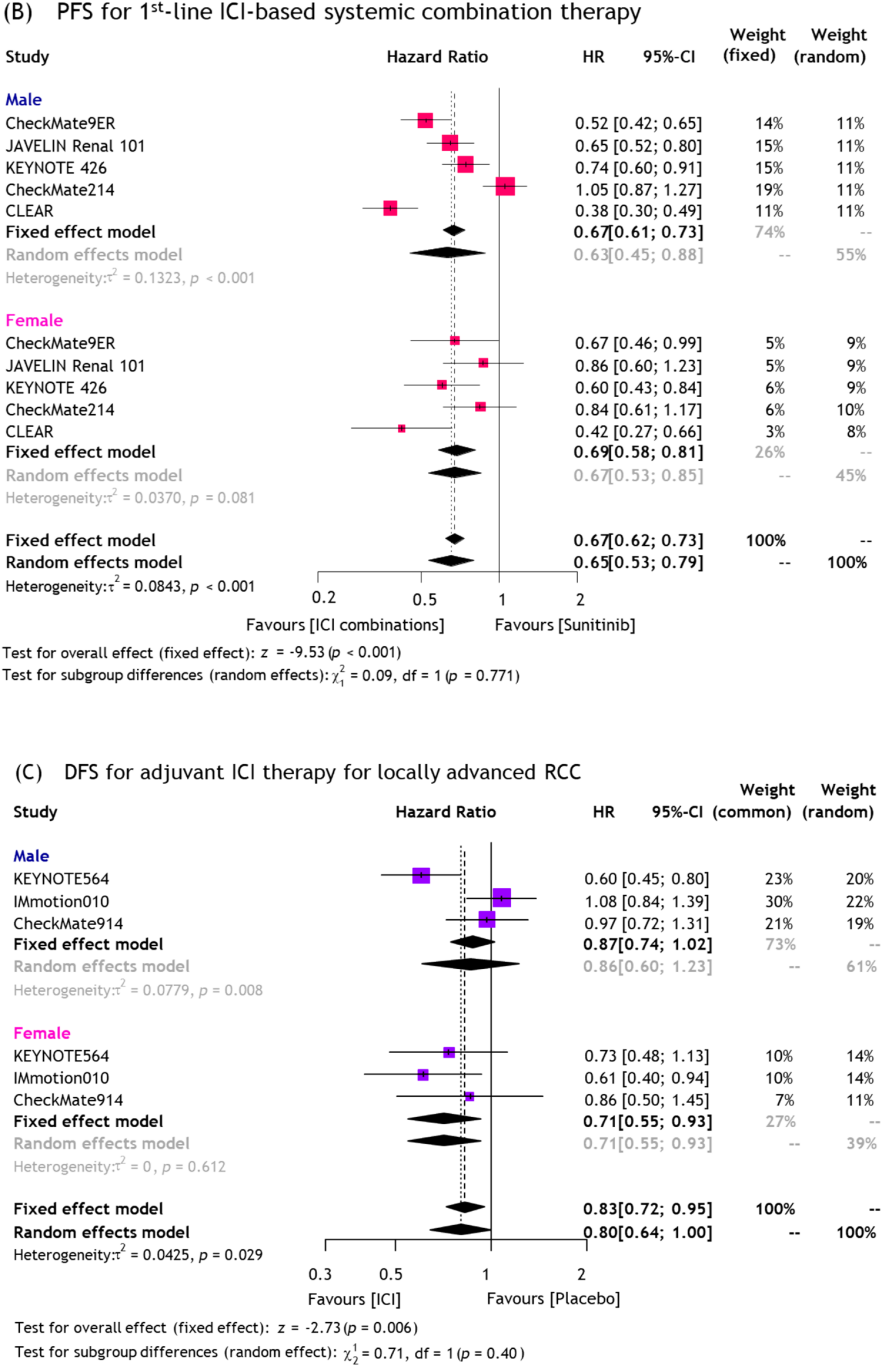
Forest plots showing association of survival outcomes and ICI therapy for RCC stratified by sex; OS **A** and PFS **B** for 1st-line ICI-based systemic combination therapy, and **C** DFS for adjuvant ICI therapy for locally advanced RCC

When limited to the 1st-line setting, ICI-based combination therapy significantly improved OS in both male (pooled HR: 0.76, 95%CI 0.67–0.86) and female (pooled HR: 0.63, 95%CI 0.51–0.77) patients compared to sunitinib alone; there was no statistically significant difference between the sexes (p = 0.12, Fig. 1A). There was no significant heterogeneity in these analyses.

*PFS*: In the 1st-line setting, ICI-based combination therapy also significantly reduced the risk of disease progression in both male (pooled HR: 0.63, 95%CI 0.45–0.88) and female (pooled HR: 0.69, 95%CI 0.58–0.81) patients compared to sunitinib alone, with no statistically significant difference in PFS between the sexes (p = 0.8, Fig. 1B). Cochrane’s Q tests revealed significant heterogeneity in the analysis of male patients (p < 0.001).

*ORR*: Four RCTs provided data on ORRs stratified by sex. The forest plot showed no statistical differences in ORR between male and female patients (pooled OR: 1.03, 95%CI 0.83–1.28, Supplementary Fig. 5).

##### Efficacy of adjuvant ICI therapies for locally advanced RCC

As shown in Fig. 1C, adjuvant ICI therapy significantly reduced the risk of disease recurrence in female patients (pooled HR: 0.71, 95%CI 0.55–0.93), whereas there was no statistically significant improvement in the recurrence rate in male patients (pooled HR: 0.86, 95%CI 0.60–1.23). No statistically significant differences were seen in OS between the sexes (p = 0.4). Cochrane’s Q tests revealed significant heterogeneity in the analysis of male patients (p = 0.008).

#### Network meta-analysis

##### 1st-line ICI-based combination therapies for mRCC

**Fig. 2:**
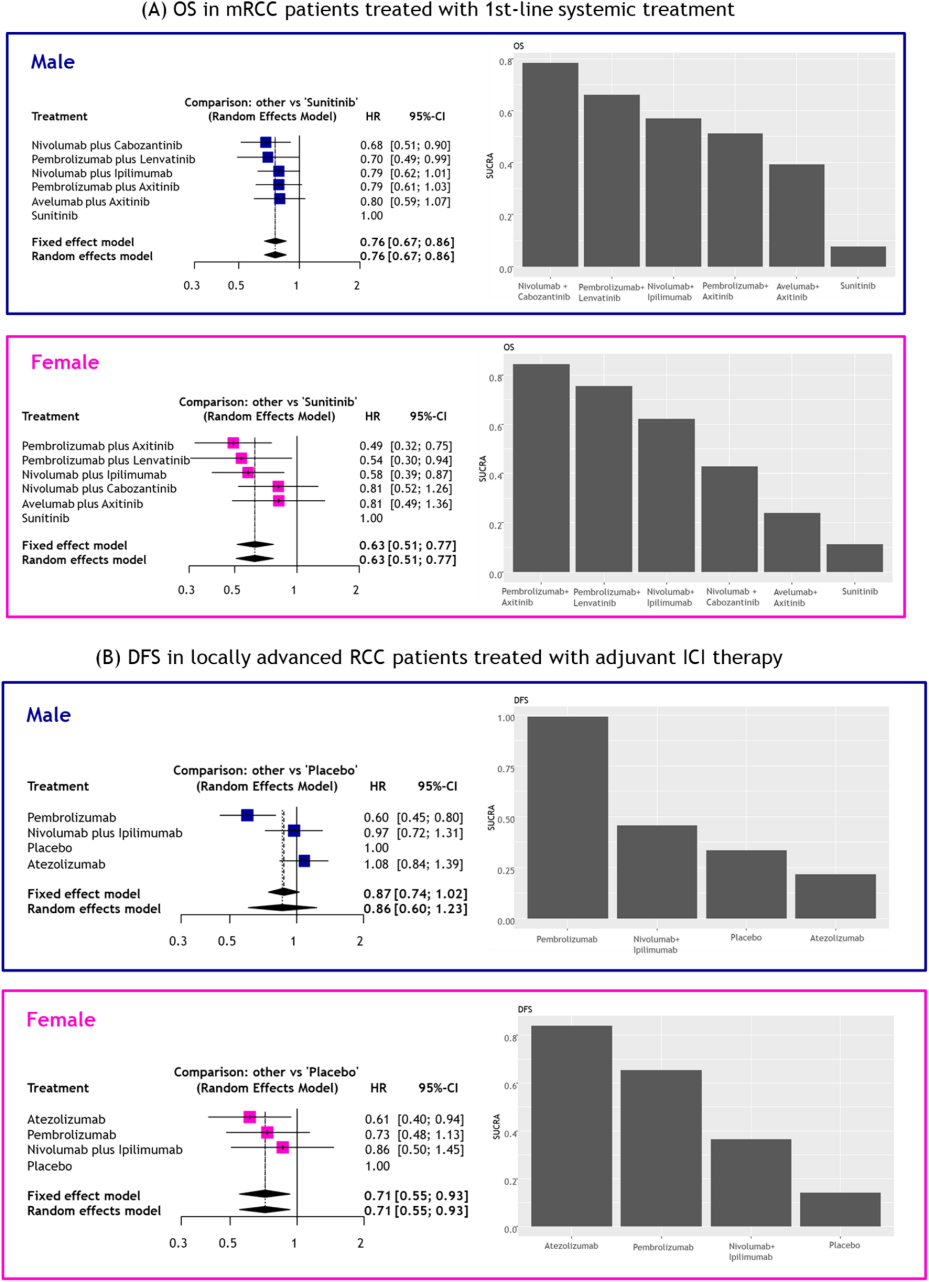
Forest plots and SUCRA graph from NMAs for **A** OS in mRCC patients treated with 1^st^-line systemic treatment and **B** DFS in locally advanced RCC patients treated with adjuvant ICI therapy

Five different ICI-based regimens, such as nivolumab + cabozantinib, avelumab + axitinib, nivolumab + ipilimumab, pembrolizumab + axitinib, and pembrolizumab + lenvatinib were included in this NMA. Network plots of all NMAs are depicted in Supplementary Fig. 6. As shown in Fig. 2, compared to sunitinib alone, nivolumab + cabozantinib (HR: 0.68, 95%CI 0.51–0.90) and pembrolizumab + lenvatinib (HR: 0.70, 95%CI: 0.49–0.99) reduced the risk of overall mortality in male patients, while pembrolizumab + axitinib (HR: 0.49, 95%CI 0.32–0.75), pembrolizumab + lenvatinib (HR: 0.54, 95%CI 0.30–0.94), and nivolumab + ipilimumab (HR: 0.58, 95%CI 0.39–0.87) reduced the risk of overall mortality in female patients. The SUCRA analysis of treatment rankings revealed that nivolumab + cabozantinib had the highest likelihood of providing the maximal OS benefit in males (78%) and pembrolizumab + axitinib had the highest likelihood of providing the maximal OS benefit in females (84%) (Fig. 2). Analysis for PFS is described in Supplementary Fig. 7.

##### Adjuvant ICI therapies for locally advanced RCC

Three different ICI-based regimens, including pembrolizumab, atezolizumab, and nivolumab + ipilimumab, were eligible for this NMA. As shown in Fig. 2, compared to placebo, only pembrolizumab (HR: 0.60, 95%CI 0.45–0.80) reduced the risk of disease recurrence in male patients, while only atezolizumab (HR: 0.61, 95%CI 0.40–0.94) reduced the risk of disease recurrence in female patients. The SUCRA analysis of treatment rankings revealed that pembrolizumab had the highest likelihood of providing the maximal DFS benefit in males (99%) and atezolizumab had the highest likelihood of providing the maximal DFS benefit in females (84%) (Fig. 2).

### UC

#### Study selection and characteristics

The study demographics and oncologic outcomes of included studies are shown in Supplementary Table 3. Of seven studies included, three studies comprising 2,240 patients assessed ICI-based combination therapy for mUC as 1st-line treatment [36, 37, 39], one study investigated pembrolizumab as 2nd-line treatment for progression after 1st line chemotherapy for mUC [35], one study for maintenance treatment after first-line chemotherapy for locally advanced or mUC [38], and two studies comprising 1518 patients evaluated ICI monotherapy as adjuvant therapy in muscle-invasive UC patients after radical surgery [4, 40].

#### Meta-analysis

##### Efficacy of ICI-based systemic therapy for mUC

Systemic therapy with ICI significantly reduced the risk of overall mortality in male patients (pooled HR: 0.80, 95%CI: 0.73–0.88) as well as female patients (pooled HR: 0.84, 95%CI: 0.70–1.00) compared to SOC (Supplementary Fig. 8). There was no significant difference between male and female patients in terms of the OS benefit with regards to systemic therapy with ICI (p = 0.7).

**Fig. 3:**
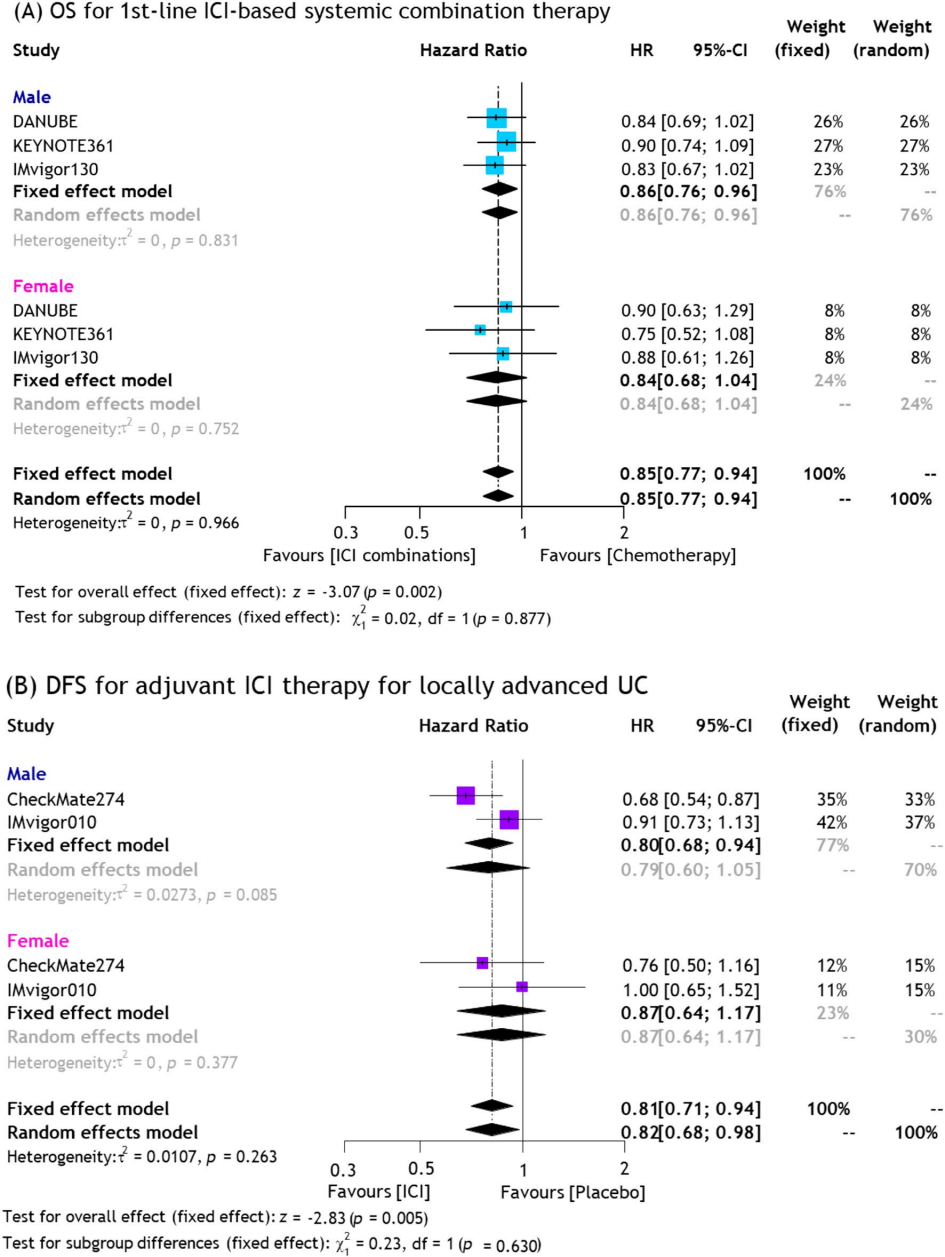
Forest plots showing association of survival outcomes and ICI therapy for UC stratified by sex; **A** OS for 1st-line ICI-based systemic combination therapy and **B** DFS for adjuvant ICI therapy for locally advanced UC

When limited to the 1st-line setting, ICI-based combination therapy significantly reduced the risk of overall mortality in male (pooled HR: 0.86, 95%CI: 0.76–0.96), but not in female (pooled HR: 0.84, 95%CI: 0.68–1.04) patients, compared to chemotherapy (Fig. 3). There were no statistically significant differences in OS between sexes (p = 0.9, Fig. 3A). We did not find any significant heterogeneity in these analyses.

##### Efficacy of adjuvant ICI therapies for locally advanced UC

Adjuvant ICI therapy significantly reduced the risk of disease recurrence in male (pooled HR: 0.80, 95%CI: 0.68–0.94), but not in female (pooled HR: 0.87, 95%CI: 0.64–1.17) patients, compared to placebo or observation (Fig. 3B). No statistically significant differences were observed in DFS between sexes (p = 0.6). We did not find significant heterogeneity in the analyses.

#### Network meta-analysis of 1st-line ICI-based combination therapies for mUC

Three different ICI-based regimens, including atezolizumab + chemotherapy, durvalumab + tremelimumab, and pembrolizumab + chemotherapy, were eligible for this NMA. The SUCRA analysis of treatment rankings revealed that atezolizumab + chemotherapy (77%) in males and pembrolizumab + chemotherapy (81%) in females to provide the highest likelihood of maximal OS benefit (Supplementary Fig. 9).

## Discussion

In this meta-analysis and NMA, we comprehensively evaluated the differential impact of sex on oncologic outcomes in both RCC and UC patients treated with ICI-based treatment. We found several key findings. First, in the first-line treatment for mRCC and mUC patients, ICI-based combination therapies significantly improved OS compared to the current standard of care regardless of sex. Second, adjuvant ICI monotherapy significantly reduced the risk of disease recurrence in female patients with locally advanced RCC and in male patients with muscle-invasive UC, whereas the reverse did not reach statistical significance. Third, treatment ranking analyses in each clinical setting of RCC and UC showed different results between sexes.

Sex-dependent immune responses are an emerging area of research [9, 10]. For example, sex hormones and X chromosome number seem to be associated with type-1 interferon (IFN-1) response [10]. The pathway relating to host defense, which is orchestrated by IFN-1, displays different activity between sexes and potentially contributes to differences in immune responses to immunotherapy between the sexes [9]. In the context of pan-cancer analyses of ICI therapy, sex has been reported as an important variable in determining response to treatment, with a trend to inferior response in female patients [13, 41]. Explanation for the observed disparity between sexes in the response to ICI therapy includes differences in the expression of programmed death-ligand 1 (PD-L1) which is partly regulated by estrogen [42]. However, despite these hypotheses and previous findings, our analyses revealed no significant differences between sexes in the efficacy of ICI-based systemic therapy for mRCC and mUC.

In the 1st-line mRCC setting, our meta-analysis revealed that ICI-based combination therapies reduced the risk of death by 24% in male and 37% in female patients, compared to sunitinib alone. The difference between sexes did not reach statistical significance (p = 0.12). Despite this lack of statistical significance, females seem to have a larger benefit of ICI therapy in mRCC as well as the adjuvant RCC setting. This disparity in survival outcomes in RCC patients between sexes could be related to genetic, hormonal, and/or social (i.e., behavioral) differences. Tulchiner et al. found an increase in estradiol and luteinizing hormone (LH)/ follicle-stimulating hormone (FSH) ratio in male patients during nivolumab monotherapy for mRCC; they also reported that the increased LH/FSH ratio was associated with worse PFS and ORR [43]. Sex disparities in oncologic outcomes in mRCC patients remain controversial.

In mRCC patients, our NMAs showed sex-specific differential treatment rankings in which nivolumab + cabozantinib had the highest likelihood of reduced risk of overall mortality in males, while pembrolizumab + axitinib had the highest likelihood in females. Interestingly, nivolumab + cabozantinib ranked fourth in female patients, and pembrolizumab + axitinib also ranked fourth in male patients. Moreover, in a recently published NMA, pembrolizumab + lenvatinib had the highest probability of being the best treatment in terms of OS among all mRCC patients [44]. In addition, in the adjuvant setting, despite atezolizumab not showing a DFS benefit in the entire cohort [32], atezolizumab significantly reduced the risk of disease progression in females compared to placebo. Even though a rationale for these different efficacies was not evaluated, our results might help improve clinical decision-making and personalizing treatment allocation according to the sex. Further investigations of different cancer states with combination regimens are warranted to obtain a definitive supporting rationale for the sex disparity regarding the efficacy of ICI-based therapy for RCC.

In urothelial carcinoma of the bladder (UCB), sex-related differences in the incidence, etiology, and response to immunotherapy are well documented [45]. In muscle-invasive UCB patients, the sex disparity in survival outcomes was demonstrated in recent meta-analyses, while the differential outcomes were not seen in non-muscle invasive bladder cancer (NMIBC) or upper tract UC [6, 7]. The studies concluded that female sex is associated with worse survival outcomes, including cancer-specific and OS [6, 7]. In addition, in the context of immunotherapy for UCB, sex differences are known with regards to response to intravesical Bacillus Calmette-Guérin (BCG) which is used for the treatment of NMIBC [46]. Regarding systemic immunotherapy for UC, our analysis revealed that adjuvant ICI monotherapy significantly reduced the risk of disease recurrence in male patients with locally advanced UC following radical surgery, whereas risk reduction did not reach statistical significance in female patients. This is in line with recent evidence suggesting that estrogens contribute to increased PD-L1 expression [42]. However, in mUC patients, despite worse prognosis and immune response to ICI in females based on previous evidence, our analysis revealed that first-line ICI-based combination therapies reduced the risk of death by the same margin in male and female patients (14% and 16%, respectively) compared to chemotherapy alone. Several biological sex disparities, such as the protective role of estrogen against carcinogenesis or enrichment of basal subtype in females have been reported [45]. Further investigations, specifically in UCB patients treated with ICI-based systemic therapy, are warranted.

In mUC patients, a recent meta-analysis revealed that ICI-based combination therapy significantly reduced the risk of death in the entire cohort and atezolizumab + chemotherapy had the highest likelihood of providing the maximal OS [47]. Interestingly, our differential treatment ranking depending on patients’ sex indicated that atezolizumab + chemotherapy had the highest likelihood of reducing the risk of overall mortality in males, while pembrolizumab + chemotherapy had the highest in females. Despite the limitation of fewer female patients as well as restrictions related to subgroup analysis, our findings might help guide clinical decision-making. Again, more investigations are needed to obtain the rationale for the differences.

The present study has several limitations that need to be considered. First, included RCTs differed in patient populations, such as the proportion of disease, burden, as well as the type of sequential therapies. Second, our analyses were performed based on subgroup analyses of each RCT, therefore sometimes suffering from a limited number of patients. Indeed, fewer female patients were included in all studies with approximately 25% of included patients being female in both RCC and UC studies. Third, for the metastatic setting, most trials assessed ICI-based combination regimen. Therefore, sex differences in efficacy cannot be attributed to ICI alone. Fourth, NMAs have a limited role in facilitating proper patient selection for current treatment options; this approach cannot substitute a direct comparison of each treatment. Finally, other than immune response, anatomical, genetic, and/or hormonal differences can influence outcomes and tumor behaviors. Further investigation of the multifactorial origin of sex-related disparities in the incidence and outcomes of UC and RCC is needed to facilitate a step forward towards personalized medicine in the era of immune therapy.

## Conclusions

In mRCC and mUC patients, OS benefit from 1st-line ICI-based combination therapy was comparable, regardless of sex. Our treatment ranking analyses showed different ICI-based regimens to be the preferred according to patient sex and clinical setting, suggesting that recommendations of ICI-based regimens considering the sex might help guide clinical decision-making. Further investigation into potential sex disparities in the immune response to ICI is needed to select the patients most likely to benefit from a specific ICI-based combination therapy. There is no doubt that sex remains an important determinant in the choice and outcome of urologic oncologic therapies.

## Supplementary Information

Below is the link to the electronic supplementary material.

Supplementary file1 (DOCX 678 KB)

## Data Availability

The data that support the findings of this study are available from the corresponding author upon reasonable request.

## Abbreviations

BCG: Bacillus Calmette-Guérin
CIs: Confidence intervals
DFS: Disease-free survival
FSH: Follicle-stimulating hormone
ICI: Immune checkpoint inhibitor
IFN-1: Type 1 interferon
IMDC: International Metastatic RCC Database Consortium
LH: Luteinizing hormone
mRCC: Metastatic renal cell carcinoma
mTOR: Mechanistic target of rapamycin
mUC: Metastatic urothelial carcinoma
NMA: Network meta-analysis
NMIBC: Non-muscle invasive bladder cancer
ORR: Objective response rate
OS: Overall survival
PD-L1: Programmed death-ligand 1
PFS: Progression-free survival
RCT: Randomized controlled trial
RCC: Renal cell carcinoma
SOC: Standard of care
SUCRA: Surface under the cumulative ranking
TKI: Tyrosine kinase inhibitor
UC: Urothelial carcinoma
UCB: Urothelial carcinoma of bladder
