# Supplementary material for: Impact of sex on the efficacy of immune checkpoint inhibitors in kidney and urothelial cancers: a systematic review and meta-analysis
Source: World J Urol. 2023 May 20;41(7):1763–74. doi: 10.1007/s00345-023-04412-0 (PMC10352444; doi:10.1007/s00345-023-04412-0)
Supplement: Supplementary file 1 — Supplementary file1 (DOCX 678 KB) [file 345_2023_4412_MOESM1_ESM.docx]

**Supplementary Information**

**1. Supplementary Appendix**. Search strategy for meta-analysis

**2. Supplementary Table 1.** PRISMA checklist 2009

**3. Supplementary Table 2.** Study demographics and oncologic outcomes of included RCTs of ICIs for RCC

**4. Supplementary Table 3.** Study demographics and oncologic outcomes of included RCTs of ICIs for UC

**5. Supplementary Table 4.** Summary of results of meta-analysis and network meta-analysis

**6. Supplementary Figure 1.** Risk of bias assessment of the included RCTs

**7. Supplementary Figure 2.** Funnel plots of pooled HRs of ICI therapy for RCC or UC; (A) OS for mRCC, (B) PFS for mRCC, (C) ORR for mRCC, (D) DFS for locally advanced RCC, (E) OS for mUC, (F) DFS for locally advanced UC

**8. Supplementary Figure 3.** The Preferred Reporting Items for Systematic Reviews and Meta-analyses (PRISMA) flow chart, detailing the article selection process

**9. Supplementary Figure 4.** Forest plots showing the association of ICI-based systemic therapy for mRCC in terms of OS including all studies.

**10. Supplementary Figure 5.** Forest plots showing the association of 1^st^-line ICI-based combination therapy for mRCC and sex in terms of ORR.

**11. Supplementary Figure 6.** Network plots showing the association of ICI-based systemic therapy for OS and PFS in mRCC patients as 1^st^-line treatment (A), DFS in locally advanced RCC as adjuvant treatment (B), and OS in mUC patients as 1^st^ line treatment (C).

**12. Supplementary Figure 7.** Forest plots and SUCRA graph from NMAs for PFS in mRCC patients treated with 1st-line systemic treatment; (A) male (B) female

**13. Supplementary Figure 8**. Forest plots showing the association of ICI-based systemic therapy for mUC in terms of OS including all studies.

**14. Supplementary** **Figure 9.** Forest plots and SUCRA graph from NMAs for OS in mUC patients treated with 1^st^-line systemic treatment; (A) male (B) female

**Abbreviations:**

PRISMA: Preferred Reporting Items for Meta-Analyses of Observational Studies in Epidemiology Statement, RCT: Randomized controlled trial, mRCC: metastatic renal cell carcinoma, mUC: metastatic urothelial carcinoma, ORR: Objective response rate, OS: Overall survival, PFS: Progression-free survival, NMA: Network meta-analysis, SUCRA: surface under the cumulative ranking

**1. Supplementary Appendix**. Search strategy for meta-analysis

Electronic searches were performed in the following databases to identify eligible studies

1. SCOPUS (n=2,568)

2. Web of science (n=1,520)

3. PubMed (n=566)

There were no language or publication period limitations.

SCOPUS

TITLE-ABS-KEY ( ( ( renal AND cell AND carcinoma ) OR ( kidney AND cancer ) OR ( urothelial AND carcinoma ) OR ( bladder AND cancer ) ) AND ( ( random ) OR ( randomized ) OR ( randomly ) ) AND ( ( immunotherapy ) OR ( immune AND checkpoint AND inhibitor ) OR ( nivolumab ) OR ( ipilimumab ) OR ( pembrolizumab ) OR ( atezolizumab ) OR ( avelumab ) OR ( tremelimumab ) ) )

Web of science

#1 (((TS=(renal cell carcinoma)) OR TS=(kidney cancer)) OR TS=(urothelial carcinoma)) OR TS=(bladder cancer)

#2 (((((((TS=(immunotherapy)) OR TS= (immune checkpoint inhibitor)) OR TS=(nivolumab)) OR TS=(ipilimumab)) OR TS=(pembrolizumab)) OR TS=(atezolizumab)) OR TS=(avelumab)) OR TS=(tremelimumab)

#3 ((TS=(random)) OR TS=(randomized)) OR TS=(randomly)

#4 #1 AND #2 AND #3 AND #4

PubMed

#1 Search: (((renal cell carcinoma[Title/Abstract]) OR (kidney cancer[Title/Abstract])) OR (urothelial carcinoma[Title/Abstract])) OR (bladder cancer[Title/Abstract]) Sort by: Most Recent

#2 Search: ((random[Title/Abstract]) OR (randomized[Title/Abstract])) OR (randomly[Title/Abstract]) Sort by: Most Recent

#3 Search: (((((((immunotherapy[Title/Abstract]) OR (immune checkpoint inhibitor[Title/Abstract])) OR (nivolumab[Title/Abstract])) OR (ipilimumab[Title/Abstract])) OR (pembrolizumab[Title/Abstract])) OR (atezolizumab[Title/Abstract])) OR (avelumab[Title/Abstract])) OR (Tremelimumab[Title/Abstract]) Sort by: Most Recent

#4 Search: ((#1) AND (#2)) AND (#3) Sort by: Most Recent

**2. Supplementary Table 1.** PRISMA checklist 2009

| **Section/topic** | **#** | **Checklist item** | **Reported on page #** |
| --- | --- | --- | --- |
| **TITLE** | | | |
| Title | 1 | Identify the report as a systematic review, meta-analysis, or both. | 1 |
| **ABSTRACT** | | | |
| Structured summary | 2 | Provide a structured summary including, as applicable: background; objectives; data sources;  study eligibility criteria, participants, and interventions; study appraisal and synthesis methods;  results; limitations; conclusions and implications of key findings; systematic review registration number. | 2 |
| **INTRODUCTION** | | | |
| Rationale | 3 | Describe the rationale for the review in the context of what is already known. | 3 |
| Objectives | 4 | Provide an explicit statement of questions being addressed with reference to participants, interventions, comparisons, outcomes, and study design (PICOS). | 4 |
| **METHODS** | | | |
| Protocol and registration | 5 | Indicate if a review protocol exists, if and where it can be accessed (e.g., Web address), and, if available, provide registration information including registration number. | 4,5 |
| Eligibility criteria | 6 | Specify study characteristics (e.g., PICOS, length of follow-up) and report characteristics  (e.g., years considered, language, publication status) used as criteria for eligibility, giving rationale. | 4,5 |
| Information sources | 7 | Describe all information sources (e.g., databases with dates of coverage, contact with study authors  to identify additional studies) in the search and date last searched. | 4,5 |
| Search | 8 | Present full electronic search strategy for at least one database, including any limits used, such that it could be repeated. | 4,5 |
| Study selection | 9 | State the process for selecting studies (i.e., screening, eligibility, included in systematic review, and,  if applicable, included in the meta-analysis). | 4,5 and Figure.S3 |
| Data collection process | 10 | Describe method of data extraction from reports (e.g., piloted forms, independently, in duplicate)  and any processes for obtaining and confirming data from investigators. | 5 |
| Data items | 11 | List and define all variables for which data were sought (e.g., PICOS, funding sources) and any assumptions and simplifications made. | 5 |
| Risk of bias in individual studies | 12 | Describe methods used for assessing risk of bias of individual studies (including specification of whether this was done at the study or outcome level), and how this information is to be used in any data synthesis. | 5 |
| Summary measures | 13 | State the principal summary measures (e.g., risk ratio, difference in means). | 5,6 |
| Synthesis of results | 14 | Describe the methods of handling data and combining results of studies, if done, including measures of consistency (e.g., I2) for each meta-analysis. | 5,6 |
| Risk of bias across studies | 15 | Specify any assessment of risk of bias that may affect the cumulative evidence (e.g., publication bias, selective reporting within studies). | 5,6 |
| Additional analyses | 16 | Describe methods of additional analyses (e.g., sensitivity or subgroup analyses, meta-regression), if done, indicating which were pre-specified. | 6 |
| RESULTS | | | |
| Study selection | 17 | Give numbers of studies screened, assessed for eligibility, and included in the review, with reasons for exclusions at each stage, ideally with a flow diagram. | 8, Figure S3 |
| Study characteristics | 18 | For each study, present characteristics for which data were extracted (e.g., study size, PICOS,  follow-up period) and provide the citations. | 8, Table S2,3 |
| Risk of bias within studies | 19 | Present data on risk of bias of each study and, if available, any outcome level assessment (see item 12). | 8,  Figure S1 |
| Results of individual studies | 20 | For all outcomes considered (benefits or harms), present, for each study: (a) simple summary data  for each intervention group (b) effect estimates and confidence intervals, ideally with a forest plot. | 9 to 13 |
| Synthesis of results | 21 | Present results of each meta-analysis done, including confidence intervals and measures of consistency. | 9 to 13 |
| Risk of bias across studies | 22 | Present results of any assessment of risk of bias across studies (see Item 15). | 8, Figure S1 |
| Additional analysis | 23 | Give results of additional analyses, if done (e.g., sensitivity or subgroup analyses, meta-regression  [see Item 16]). |  |
| **DISCUSSION** | | | |
| Summary of evidence | 24 | Summarize the main findings including the strength of evidence for each main outcome;  consider their relevance to key groups (e.g., healthcare providers, users, and policy makers). | 14-18 |
| Limitations | 25 | Discuss limitations at study and outcome level (e.g., risk of bias), and at review-level  (e.g., incomplete retrieval of identified research, reporting bias). | 17 |
| Conclusions | 26 | Provide a general interpretation of the results in the context of other evidence,  and implications for future research. | 18 |
| **FUNDING** | | | |
| Funding | 27 | Describe sources of funding for the systematic review and other support (e.g., supply of data);  role of funders for the systematic review. | 20 |

| 3. Supplementary Table 2. Study demographics and oncologic outcomes of included RCTs of ICIs for RCC | | | | | | | | | | | | | | | |
| --- | --- | --- | --- | --- | --- | --- | --- | --- | --- | --- | --- | --- | --- | --- | --- |
| Study name and first author | Year | Treatment  arm | Control  arm | Number of patients | | | | IMDC classification, n (%) | | | Median follow-up period, month | ORR of treatment arm | | HR (95%CI) of survival outcomes  (Treatment vs. Control) | |
|  |  |  |  | Treatment | | Control | |  |  |  |  |  |  |  |  |
|  |  |  |  | All | M/F, n (%) | All | M/F, n (%) |  | Treatment | Control |  | M | F | M | F |
| *1st line Treatment* | | | | | | | | | | | | | | | |
| CheckMate9ER,  Motzer et al. | 2022 | Nivolumab+ Cabozantinib | Sunitinib | 323 | M: 249 (77) F: 74 (23) | 328 | M: 232 (71) F: 96 (29) | Favorable Intermediate Poor | 74 (23) 188 (58) 61 (19) | 72 (22) 188 (57) 68 (21) | 32.9 | 56% | 55% | OS: 0.68 (0.51-0.9) PFS: 0.52 (0.42-0.65) | OS: 0.81 (0.52-1.26) PFS: 0.67 (0.46-0.99) |
| JAVELIN Renal 101, Motzer et al. Choueiri et al. | 2019/ 2020 | Avelumab+ Axitinib | Sunitinib | 442 | M: 316 (71.5) F: 126 (28.5) | 444 | M: 344 (77.5) F: 100 (22.5) | Favorable Intermediate Poor | 52 (19) 180 (67) 33 (12) | 60 (21) 201 (69) 24 (8.3) | 19.3 | 53% | 50% | OS: 0.8 (0.59-1.07) PFS: 0.65 (0.52-0.8) | OS: 0.81 (0.49-1.36) PFS: 0.86 (0.6-1.23) |
| KEYNOTE-426, Powles et al. | 2020 | Pembrolizumab+ Axitinib | Sunitinib | 432 | M: 308 (71) F: 124 (29) | 429 | M: 320 (75) F: 109 (25) | Favorable Intermediate Poor | 138 (32) 238 (55) 56 (13) | 131 (31) 246 (57) 52 (12) | 30.6 | 58% | 65% | OS: 0.79 (0.61-1.03) PFS: 0.74 (0.6-0.91) | OS: 0.49 (0.32-0.75) PFS: 0.6 (0.43-0.84) |
| CheckMate214, Motzer et al. | 2018/ 2019 | Nivolumab+ Ipilimumab | Sunitinib | 550 | M: 413 (75) F: 137 (25) | 546 | M: 395 (72) F: 151 (28) | Favorable Intermediate Poor | 125 (23) 334 (61) 91 (17) | 124 (23) 333 (61) 89 (16) | 32.4 | 41% | 41% | OS: 0.79 (0.62-1.01) PFS: 1.05 (0.87-1.27) | OS: 0.58 (0.39-0.87) PFS: 0.84 (0.61-1.17) |
| CLEAR, Motzer et al. | 2021 | Pembrolizumab+ Lenvatinib | Sunitinib | 355 | M: 255 (72) F: 100 (28) | 357 | M: 275 (77) F: 82 (23) | Favorable Intermediate Poor | 110 (27) 210 (64) 33 (9.3) | 124 (35) 192 (54) 37 (10) | 26.6 | 71% | | OS: 0.7 (0.49-0.99) PFS: 0.38 (0.3-0.49) | OS: 0.54 (0.3-0.94) PFS: 0.42 (0.27-0.66) |
| *2nd- or 3rd line Treatment* | | | | | | | | | | | | | | | |
| CheckMate025, Motzer et al. | 2015 | Nivolumab | Everolimus | 410 | M: 315 (77) F: 95 (23) | 411 | M: 304 (74) F: 107 (26) | *Favorable Intermediate Poor | 145 (35) 201 (49) 64 (16) | 148 (36) 203 (49) 60 (15) | ND Minimum: 14 | 25% | | OS: 0.73 (0.58-0.92) | OS: 0.84 (0.57-1.24) |
| *Adjuvant therapy* | | | | | | | | | | | | | | | |
| KEYNOTE-564, Choueiri et al. Powles et al. | 2021/ 2022 | Pembrolizumab | Placebo | 496 | M: 347 (70) F: 149 (30) | 498 | M: 359 (72) F: 139 (28) | NA | | | 24 | NA | | DFS: 0.6 (0.45-0.8) | DFS: 0.73 (0.48-1.13) |
| IMmotion010, Pal et al. | 2022 | Atezolizumab | Placebo | 390 | M: 287 (74) F: 103 (26) | 388 | M: 278 (72) F: 110 (28) | NA | | | 44.7 | NA | | DFS: 1.08 (0.84-1.39) | DFS: 0.61 (0.40-0.94) |
| CheckMate 914, Motzer et al. | 2022 | Nivolumab+ Ipilimumab | Placebo | 405 | M: 286 (71) F: 119 (29) | 411 | M: 294 (72) F: 117 (28) | NA | | | 37 | NA | | DFS: 0.97 (0.72-1.31) | DFS: 0.86 (0.50-1.45) |
| RCTs: Randomized controlled trials, ICI: Immune Checkpoint Inhibitors, RCC: Renal cell carcinoma, M: Male, F: Female, IMDC: International Metastatic RCC Database Consortium, ORR: Objective Response Rate, OS: Overall survival, PFS: Progression-Free Survival, DFS: Disease-Free Survival, HR: Hazard Ratio, CI: Confidence Interval, ND: No Data, NA: Not Applicable *Described as MSKCC risk classification | | | | | | | | | | | | | | | |

| 4. Supplementary Table 3. Study demographics and oncologic outcomes of included RCTs of ICIs for UC | | | | | | | | | | | |
| --- | --- | --- | --- | --- | --- | --- | --- | --- | --- | --- | --- |
| Study name  and first author | Year | Treatment  arm | Control  arm | Inclusion  criteria | Number of patients | | | | Median follow-up period, month | HR (95%CI) of survival outcomes  (treatment arm vs. control arm) | |
|  |  |  |  |  | Treatment | | Control | |  |  |  |
|  |  |  |  |  | All | M/F, n (%) | All | M/F, n (%) |  | M | F |
| *1st line Treatment* | | | | | | | | | | | |
| DANUBE, Powles et al. | 2020 | Durvalumab+ Tremelimumab | Chemotherapy | Locally advanced or metastatic UC | 342 | M: 256 (75) F: 86 (25) | 344 | M: 274 (80) F: 70 (20) | 41.2 | OS: 0.84 (0.69-1.02) | OS: 0.9 (0.63-1.3) |
| KEYNOTE-361, Powles et al. | 2021 | Pembrolizumab+ Chemotherapy | Chemotherapy | Locally advanced or metastatic UC | 351 | M: 272 (77) F: 79 (23) | 352 | M: 262 (74) F: 90 (26) | 31.7 | OS: 0.9 (0.74-1.1) | OS: 0.75 (0.52-1.08) |
| IMvigor130, Galsky et al. | 2020 | Atezolizumab+ Chemotherapy | Chemotherapy | Locally advanced or metastatic UC | 451 | M: 338 (75) F: 113 (25) | 400 | M: 298 (74.5) F: 102 (25.5) | 11.8 | OS: 0.83 (0.67-1.02) PFS: 0.83 (0.69-0.99) | OS: 0.88 (0.61-1.26) PFS: 0.75 (0.55-1.02) |
| *2nd line Treatment* | | | | | | | | | | | |
| KEYNOTE-045, Bellmunt et al. | 2017 | Pembrolizumab | Chemotherapy | Recurrence or progression after platinum-based chemotherapy | 270 | M: 200 (74) F: 70 (26) | 272 | M: 202 (74) F: 72 (26) | 14.1 | OS: 0.73 (0.56-0.94) | OS: 0.78 (0.49-1.24) |
| *Maintenance* | | | | | | | | | | | |
| JAVELIN Bladder 100, Powles et al. | 2020 | Avelumab | Placebo | Locally advanced or metastatic UC with no progression after first-line chemotherapy | 350 | M: 266 (76) F: 84 (24) | 350 | M: 275 (79) F: 75 (21) | 19 (more than) | OS: 0.64 (0.5-0.83) PFS: 0.6 (0.49-0.74) | OS: 0.89 (0.56-1.41) PFS: 0.69 (0.47-1.01) |
| *Adjuvant* | | | | | | | | | | | |
| CheckMate 274, Bajorin et al. | 2021 | Nivolumab | Placebo | Muscle-invasive UC patients who had undergone radical surgery | 353 | M: 265 (75) F: 88 (25) | 356 | M: 275 (77) F: 81 (23) | 20.9 | DFS: 0.68 (0.54-0.87) | DFS: 0.76 (0.5-1.16) |
| IMvigor010, Bellmunt et al. | 2021 | Atezolizumab | Placebo | Muscle-invasive UC patients who had undergone radical surgery | 406 | M: 322 (79) F: 84 (21) | 403 | M: 316 (78) F: 87 (22) | 21.9 | DFS: 0.91 (0.73-1.13) | DFS: 1.00 (0.65-1.52) |
| RCTs: Randomized controlled trials, ICI: Immune Checkpoint Inhibitors, UC: Urothelial Carcinoma, M: Male, F: Female, OS: Overall survival, PFS: Progression-Free Survival, DFS: Disease-Free Survival, HR: Hazard Ratio, CI: Confidence Interval | | | | | | | | | | | |

| 5. Supplementary Table 4. Summary of results of meta-analysis and network meta-analysis | | | | |
| --- | --- | --- | --- | --- |
|  | Meta-analysis | | | Network meta-analysis |
| *1. RCC* | | | | |
|  | M/F | OS, pooled HR (95%CI) | PFS, pooled HR (95%CI) | Treatment ranking for OS |
| All metastatic  setting | Male | 0.75 (0.67-0.84) | NA | NA |
|  | Female | 0.67 (0.56-0.80) |  |  |
| 1st line | Male | 0.76 (0.67-0.86) | 0.63 (0.45-0.88) | **①Nivo + Cabo: 78%** ②Pem + Len: 66% ③Nivo + Ipi: 57% ④Pem + Axi: 51% ⑤Ave + Axi: 39% |
|  | Female | 0.63 (0.51-0.77) | 0.69 (0.58-0.81) | **①Pem + Axi: 84%** ②Pem + Len: 66% ③Nivo + Ipi: 57% ①Nivo + Cabo: 43% ⑤Ave + Axi: 24% |
|  |  | DFS, pooled HR (95%CI) | |  |
| Adjuvant | Male | 0.86 (0.60-1.23) | | **①Pem: 99%** ②Nivo + Ipi: 46% ③Placebo: 34％ ④Atezo: 22% |
|  | Female | 0.71 (0.55-0.93) | | **①Atezo: 84%** ②Pem: 65% ②Nivo + Ipi: 37% ③Placebo: 14％ |
| *2. UC* | | | | |
|  |  | OS, pooled HR (95%CI) | | Treatment ranking for OS |
| All metastatic  setting | Male | 0.80 (0.73-0.88) | | NA |
|  | Female | 0.84 (0.70-1.00) | |  |
| 1st line | Male | 0.86 (0.76-0.96) | | **①Atezo + Chemo: 77%** ②Durva + Treme: 67% ③Pem + Chemo: 46% |
|  | Female | 0.84 (0.68-1.04) | | **①Pem + Chemo: 81%** ②Atezo + Chemo: 57% ③Durva + Treme: 34% |
|  |  | DFS, pooled HR (95%CI) | |  |
| Adjuvant | Male | 0.80 (0.68-0.94) | | NA |
|  | Female | 0.87 (0.64-1.17) | |  |
| M: Male, F: Female, RCC: Renal Cell Carcinoma, UC: Urothelial Carcinoma, OS: Overall Survival, PFS: Progression-Free Survival, DFS: Disease-Free Survival, HR: Hazard Ratio, CI: Confidence Interval, NA: Not Applicable, Nivo: Nivolumab, Cabo: Cabozantinib, Pem: Pembrolizumab, Len: Lenvatinib, Ipi: Ipilimumab, Axi: Axitinib, Ave: Avelumab, Atezo: Atezolizumab, Chemo: Chemotherapy, Durva: Durvalumab, Treme: Tremelimumab | | | | |

**6. Supplementary Figure 1.** Risk of bias assessment of the included RCTs


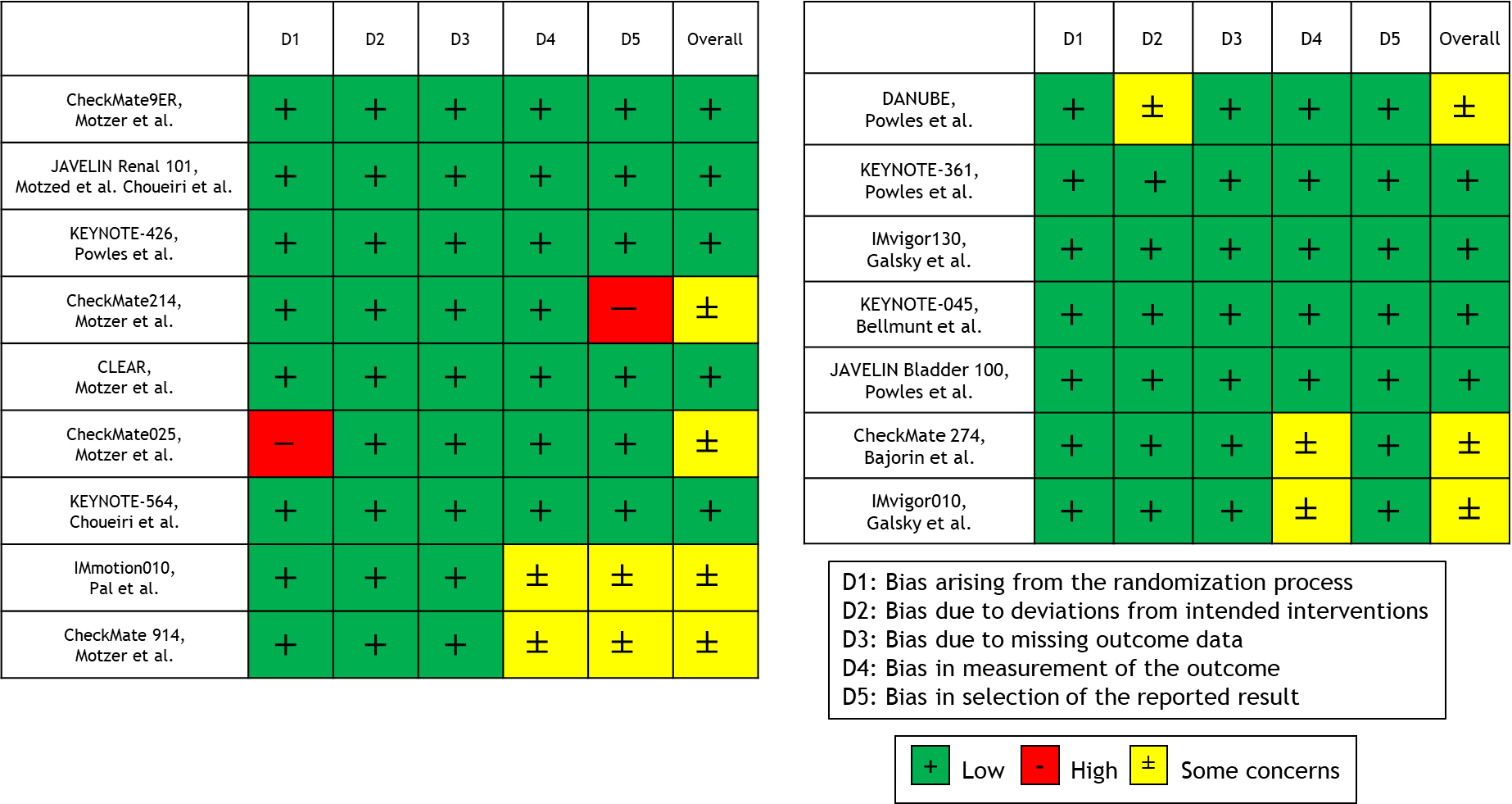


**7. Supplementary Figure 2**. Funnel plots of pooled HRs of ICI therapy for RCC or UC; (A) OS for mRCC, (B) PFS for mRCC, (C) ORR for mRCC, (D) DFS for locally advanced RCC, (E) OS for mUC, (F) DFS for locally advanced UC

(A)


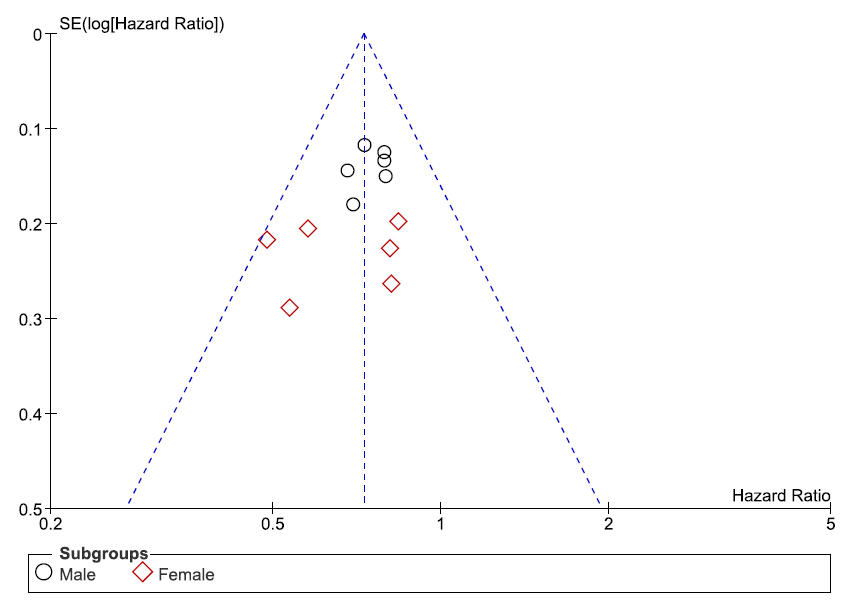


(B)


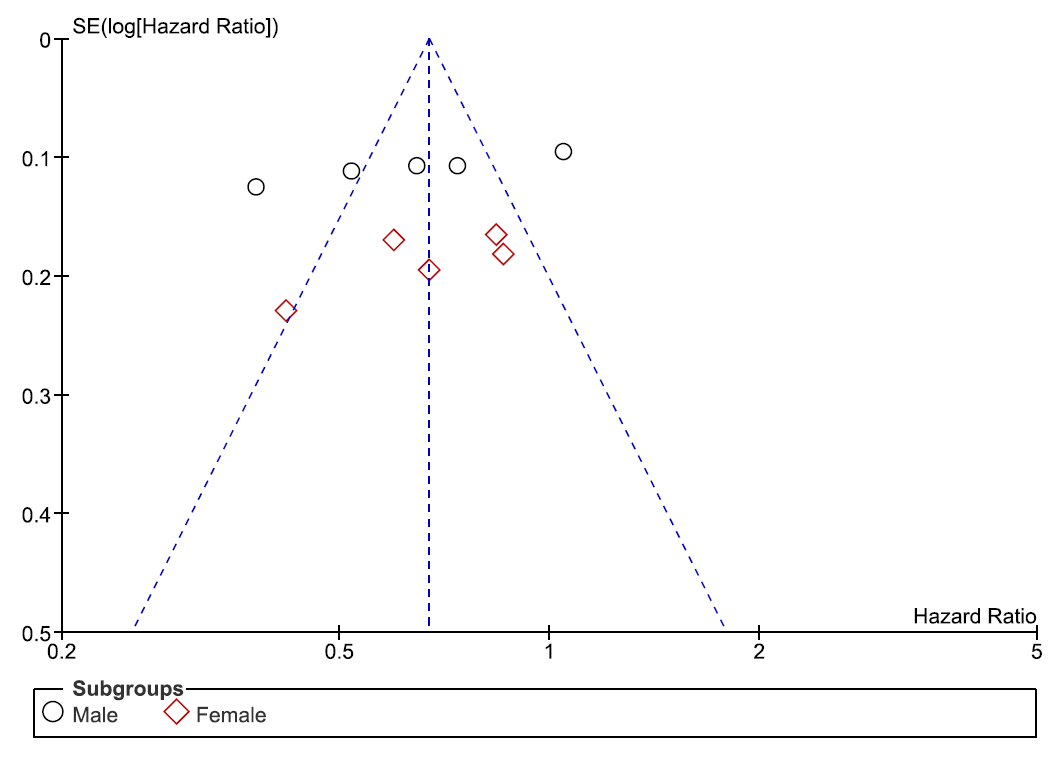


(C)


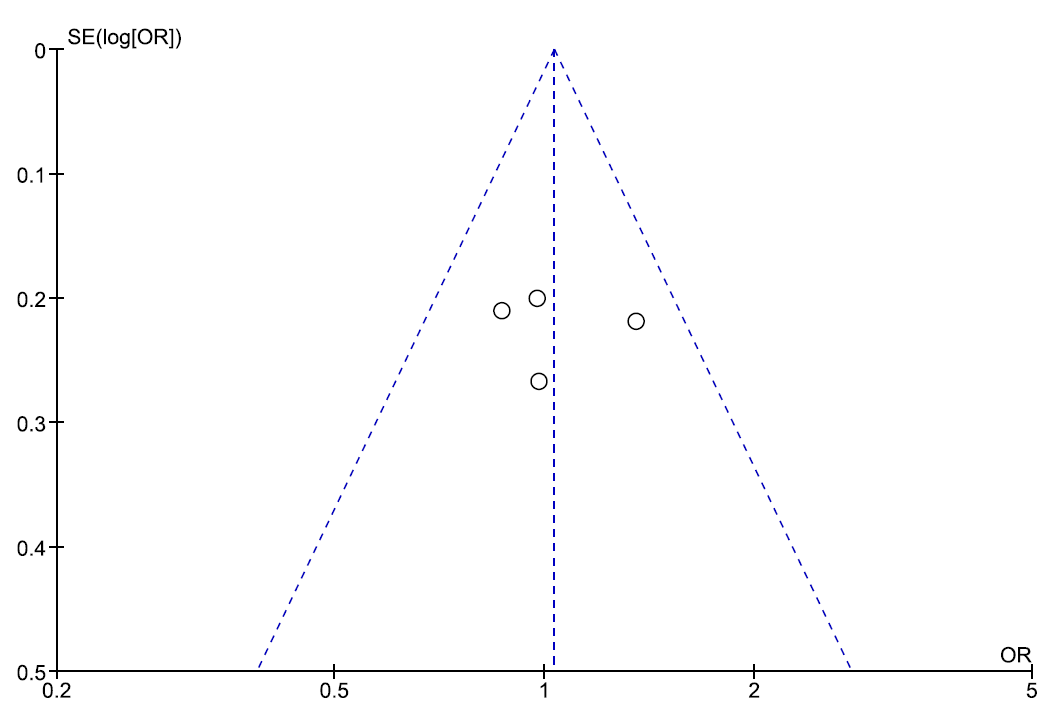


(D)


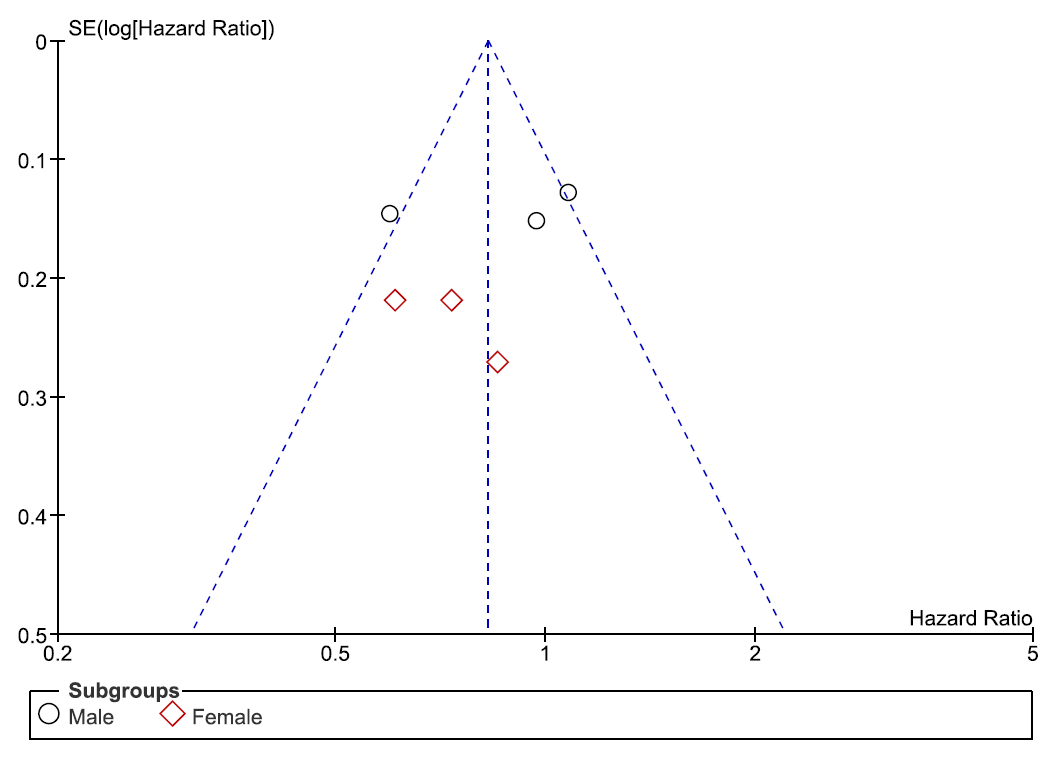


(E)


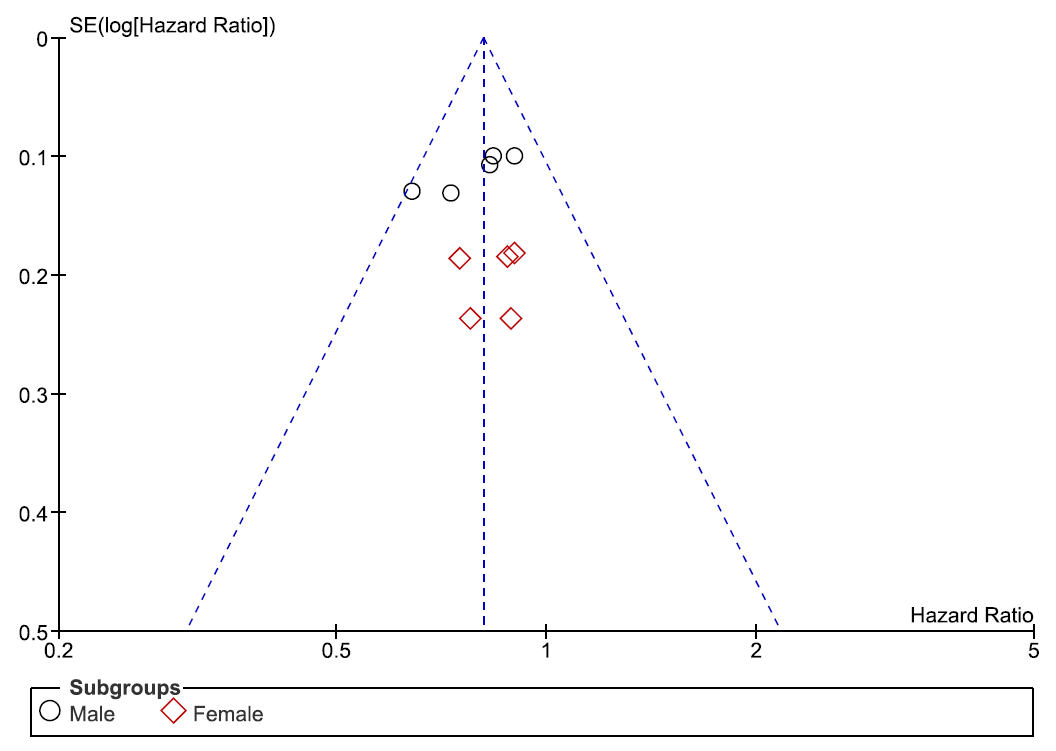


(F)


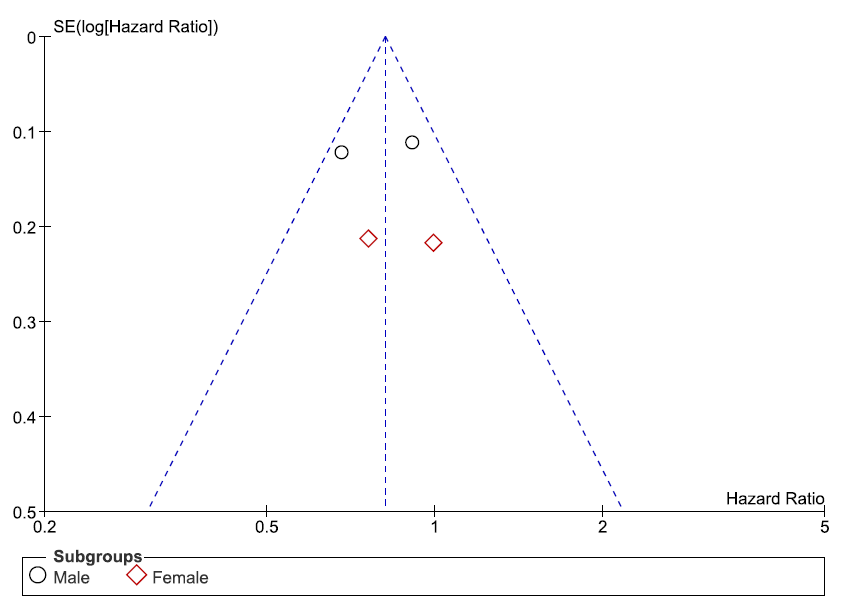


**8. Supplementary Figure 3.** The Preferred Reporting Items for Systematic Reviews and Meta-analyses (PRISMA) flow chart, detailing the article selection process

**Identification of studies via databases and registers**

**Records identified through PUBMED, Web of Science, Scopus:**

**Search Query:**

*(Renal cell carcinoma) OR (kidney cancer) OR (urothelial carcinoma) OR (bladder cancer) AND (random) OR (randomized) OR (randomly) AND (immunotherapy) OR (immune checkpoint inhibitor) OR (nivolumab) OR (ipilimumab) OR (pembrolizumab) OR (atezolizumab) OR (avelumab) OR (tremelimumab)*

(n=4,654)

**Identification**

**Records excluded after title and abstract review** (n =2,485)

・Non-relevant according to inclusion criteria (n=2,202)

・Review article (n=164)

・Letter/ Editorial comment (n=64)

・Other than English (n=55)

**Records screened after duplicates removed**

(n =2,533)

**Screening**

**Full-text articles assessed for eligibility**

(n =48)

**Records excluded after evaluation**

(n =30)

・Non-clear data regarding association between the systemic therapy and survival (n=10)

・No data of survival outcomes stratified by sex (n=4)

・Not phaseⅢ trials (n=16)

**Eligibility**

**Included**

**Studies included in meta-analysis**

(n=18 [16 RCTs])

**9. Supplementary Figure 4.** Forest plots showing the association of ICI-based systemic therapy for mRCC in terms of OS including all studies.


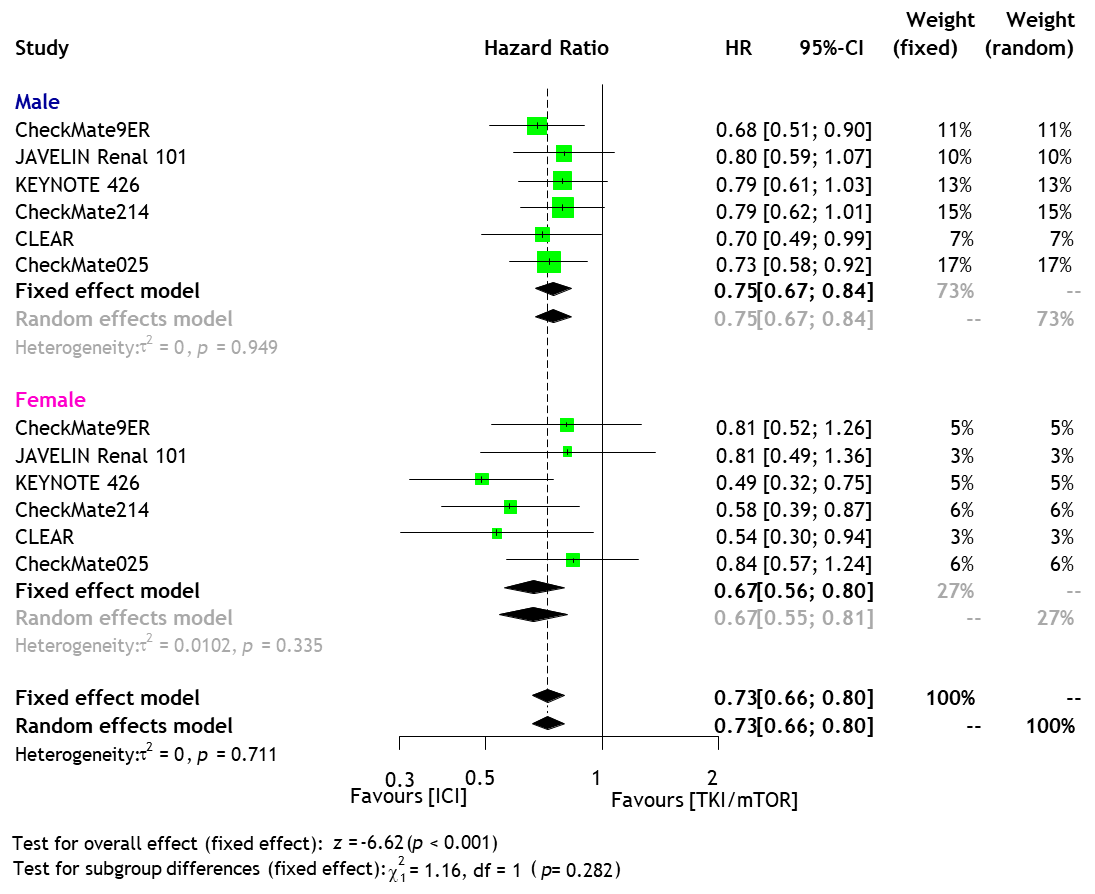


**10. Supplementary Figure 5.** Forest plots showing the association of 1^st^-line ICI-based combination therapy for mRCC and sex in terms of ORR.


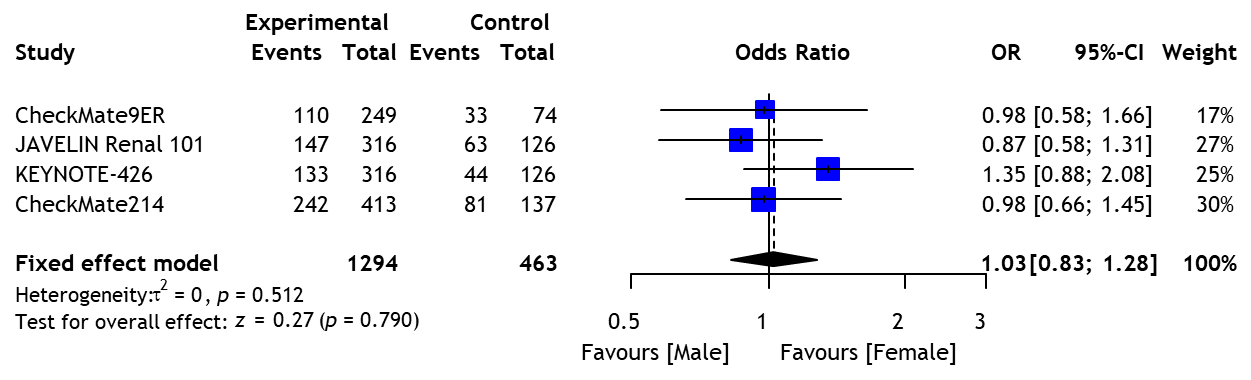


**11. Supplementary Figure 6.** Network plots showing the association of ICI-based systemic therapy for OS and PFS in mRCC patients as 1^st^-line treatment (A), DFS in locally advanced RCC as adjuvant treatment (B), and OS in mUC patients as 1^st^ line treatment (C).

(A)


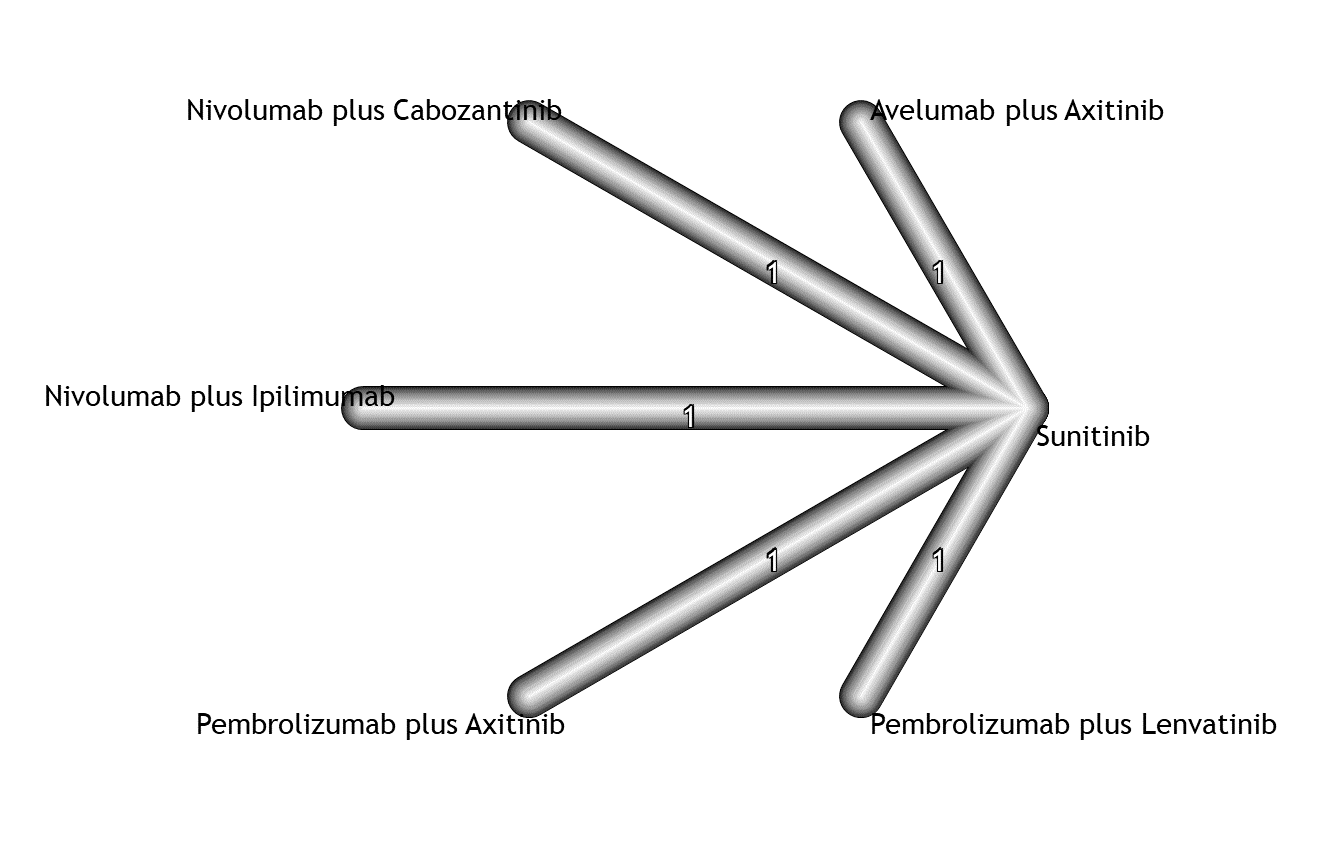


(B)


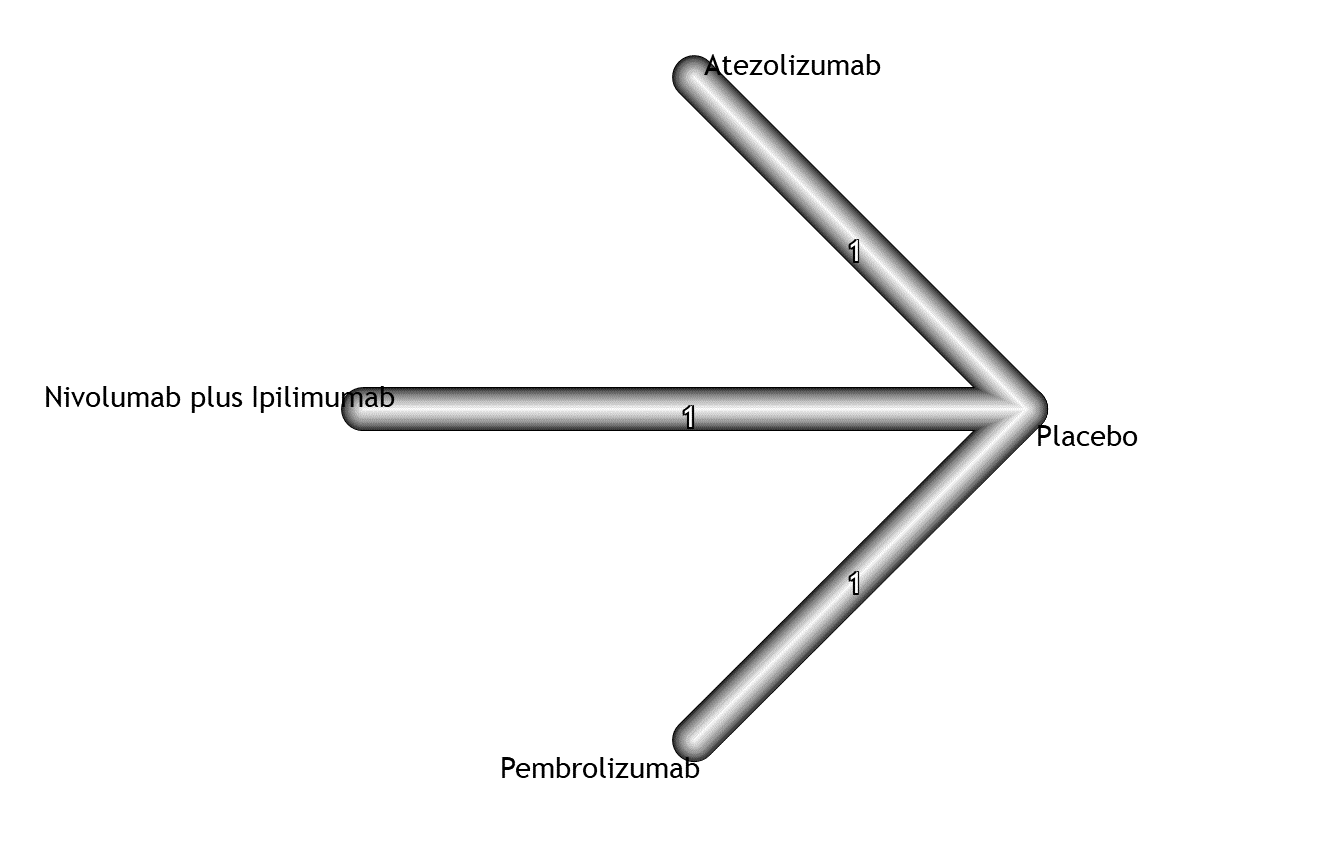


(C)


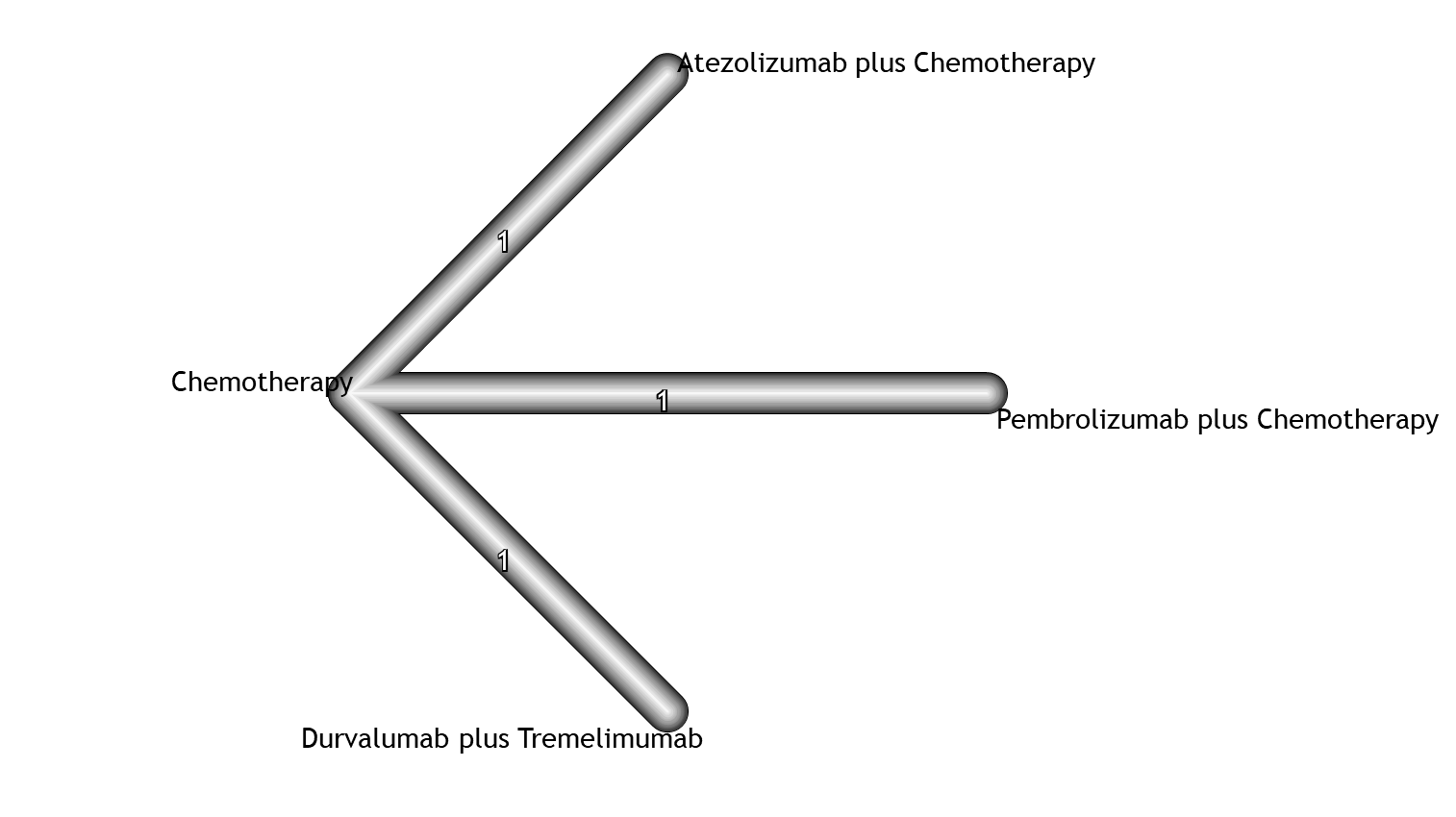


**12. Supplementary Figure 7.** Forest plots and SUCRA graph from NMAs for PFS in mRCC patients treated with 1st-line systemic treatment; (A) male (B) female

(A) Male


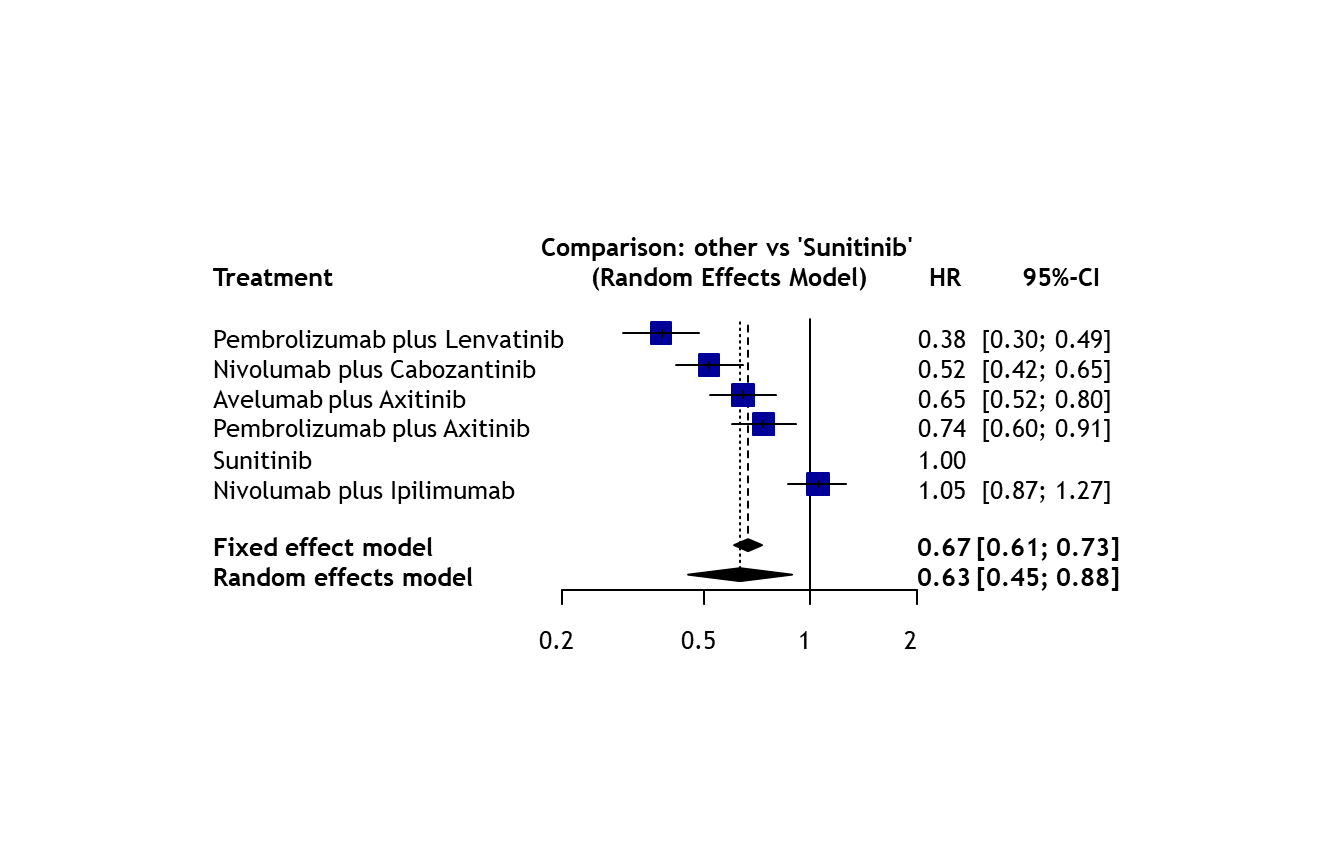

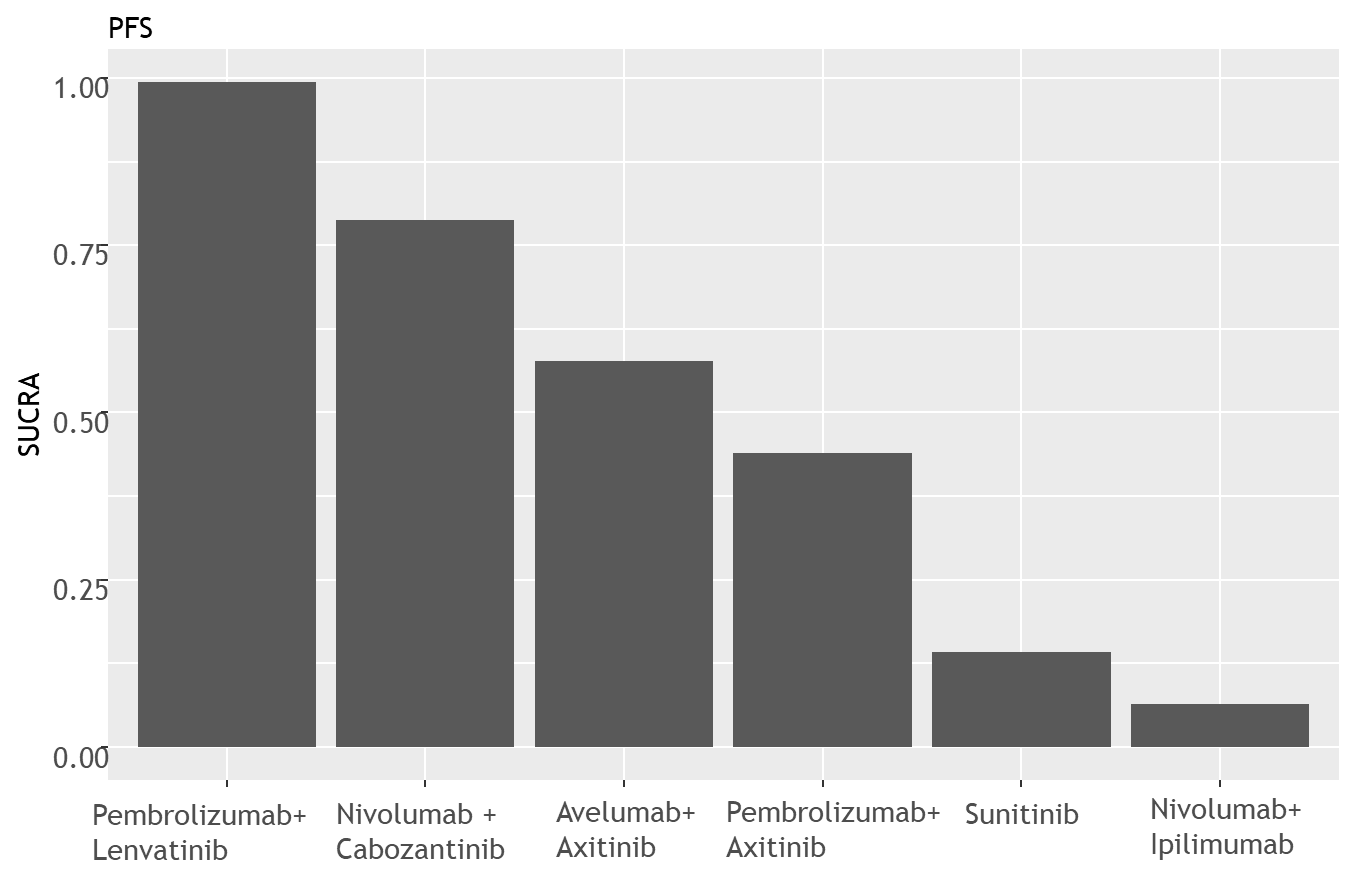


(B) Female


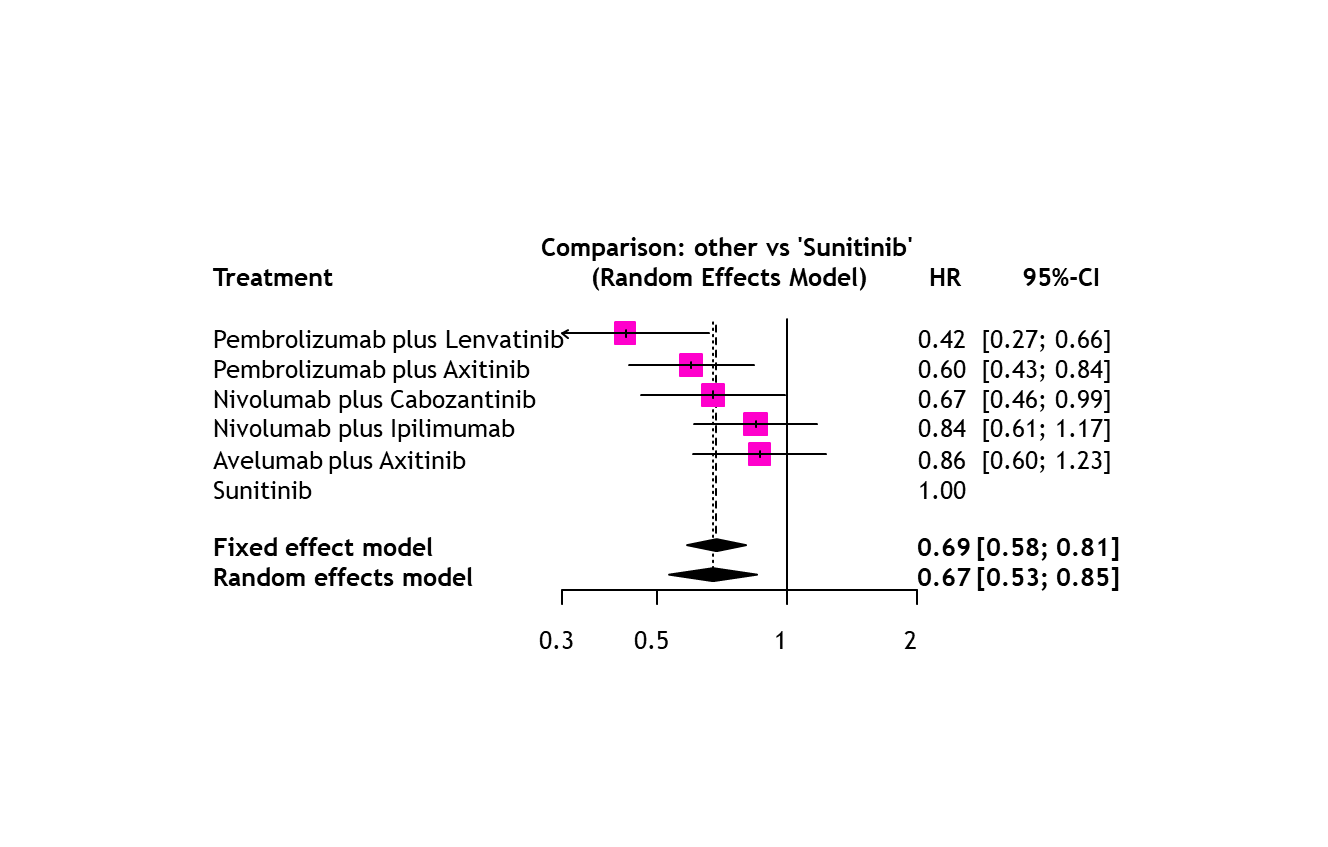

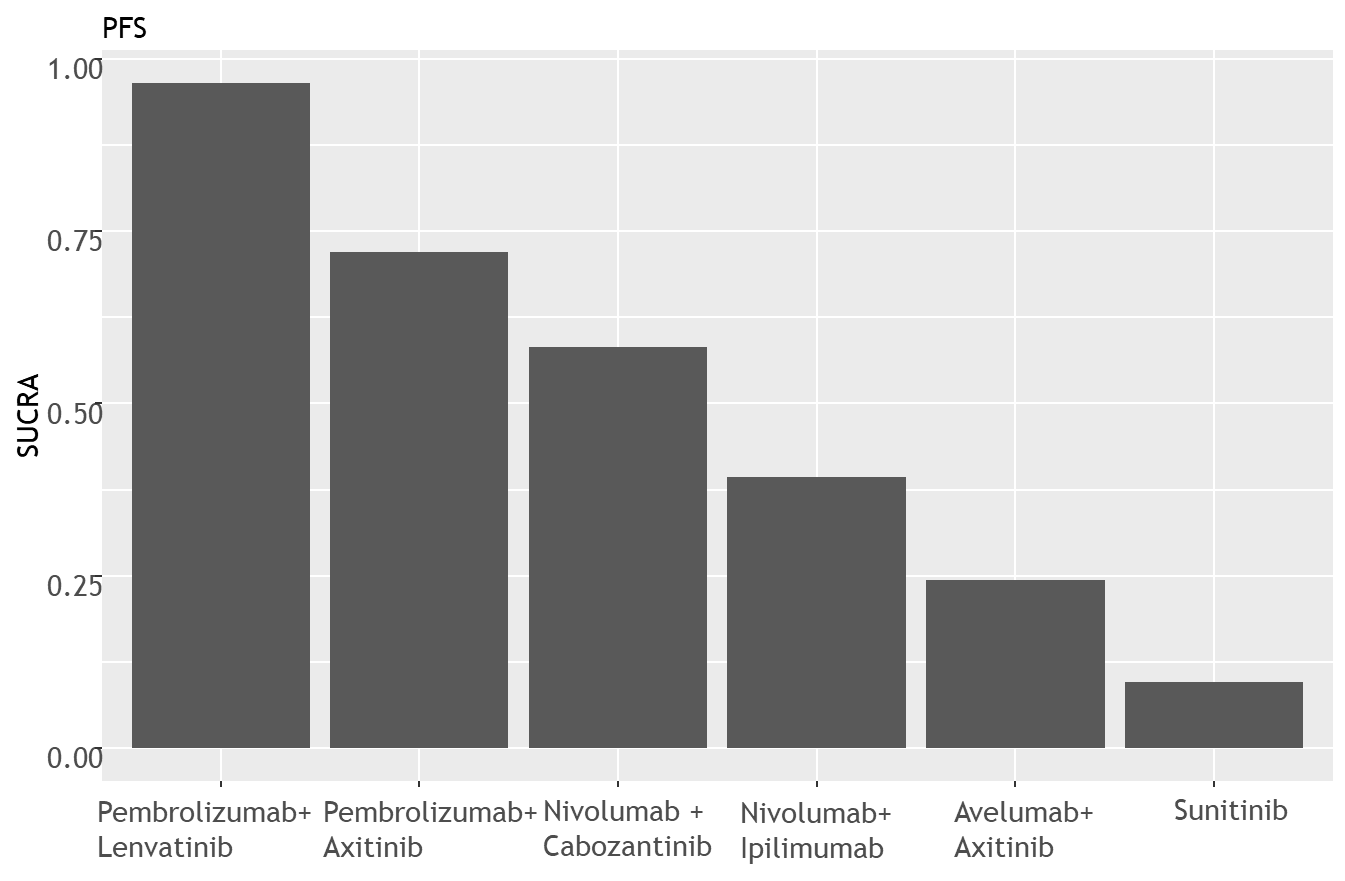


**13. Supplementary Figure 8.** Forest plots showing the association of ICI-based systemic therapy for mUC in terms of OS including all studies.


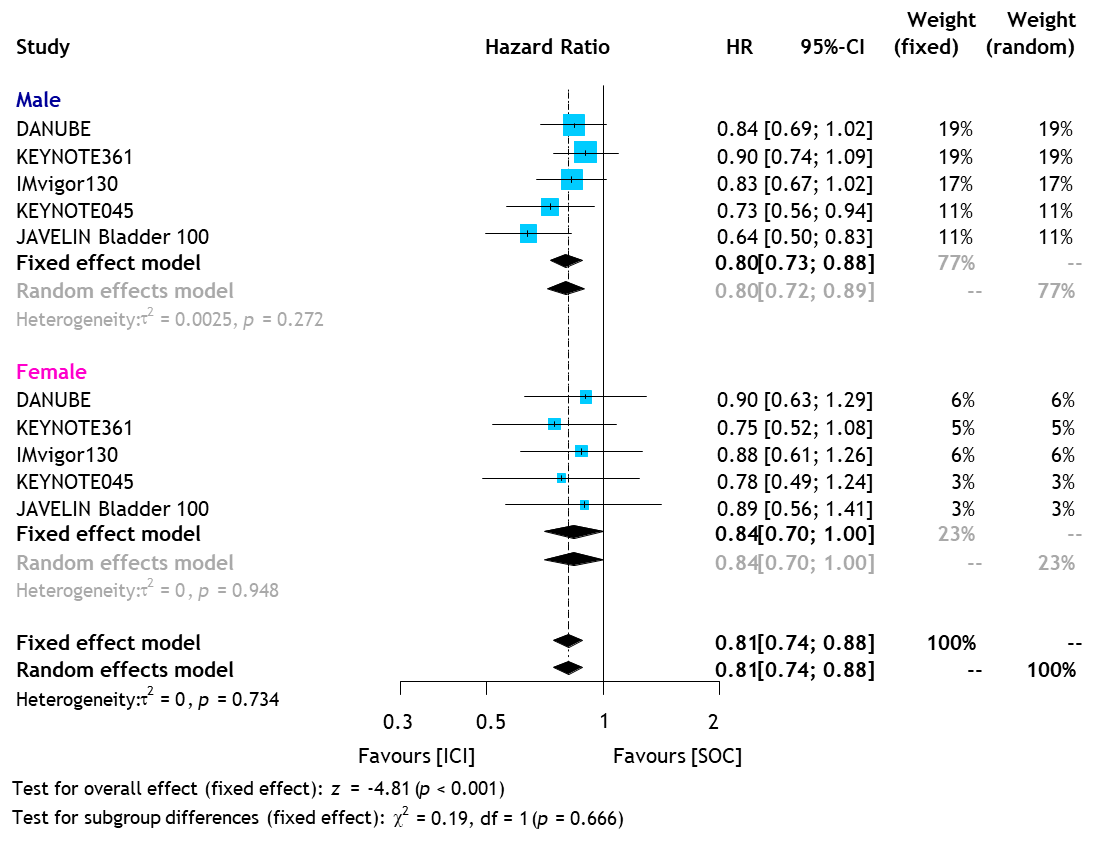


**14. Supplementary** **Figure 9.** Forest plots and SUCRA graph from NMAs for OS in mUC patients treated with 1^st^-line systemic treatment; (A) male (B) female

(A) Male


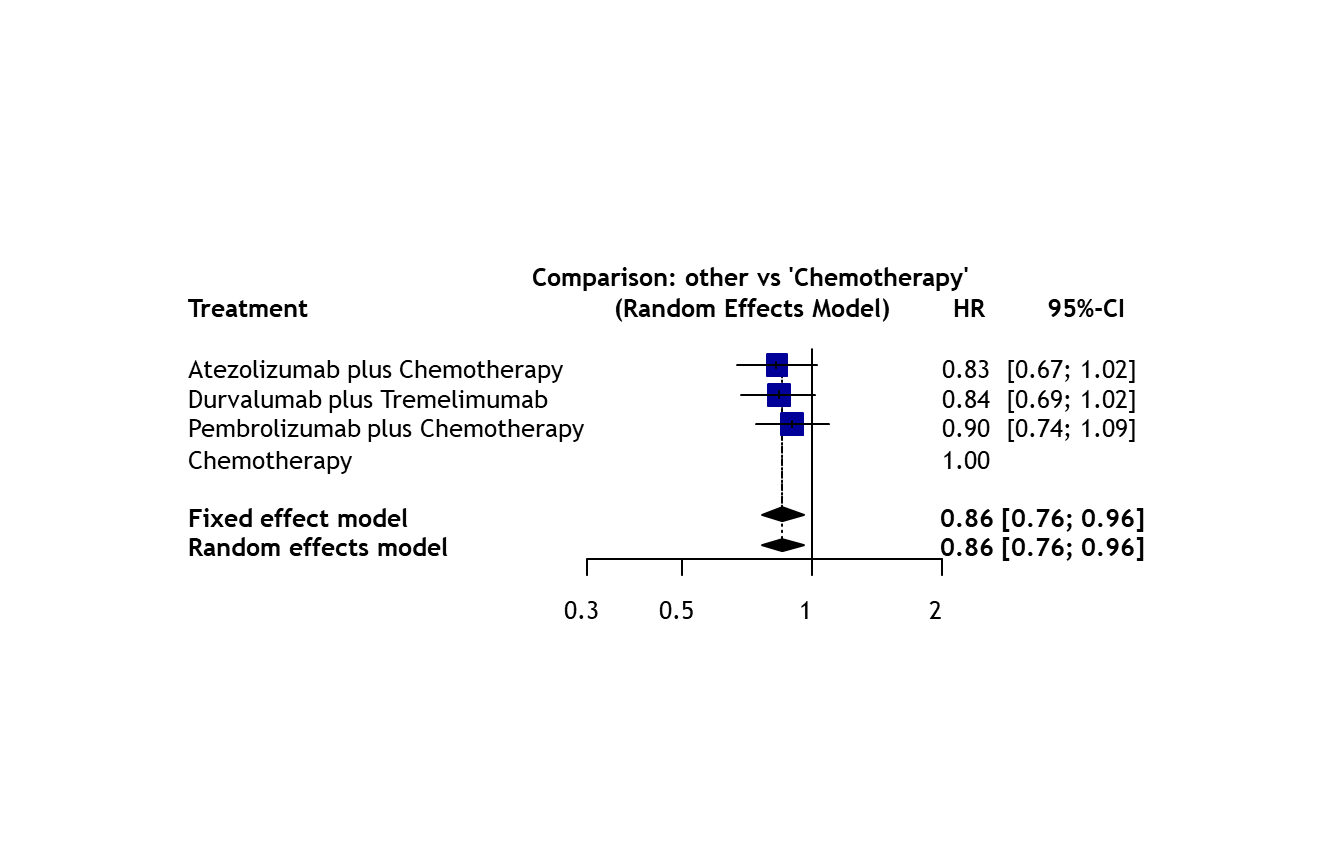

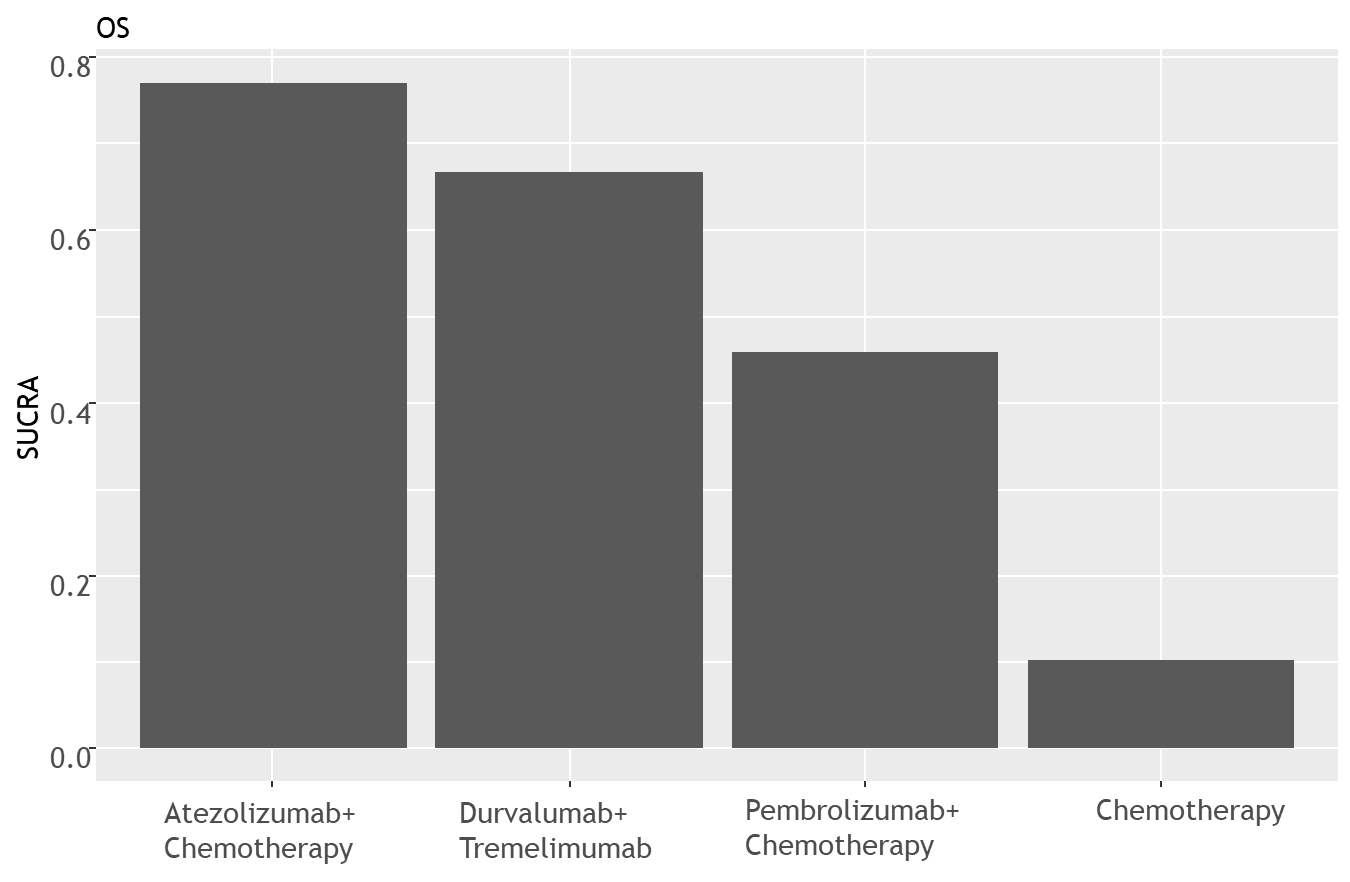


(B) Female

**
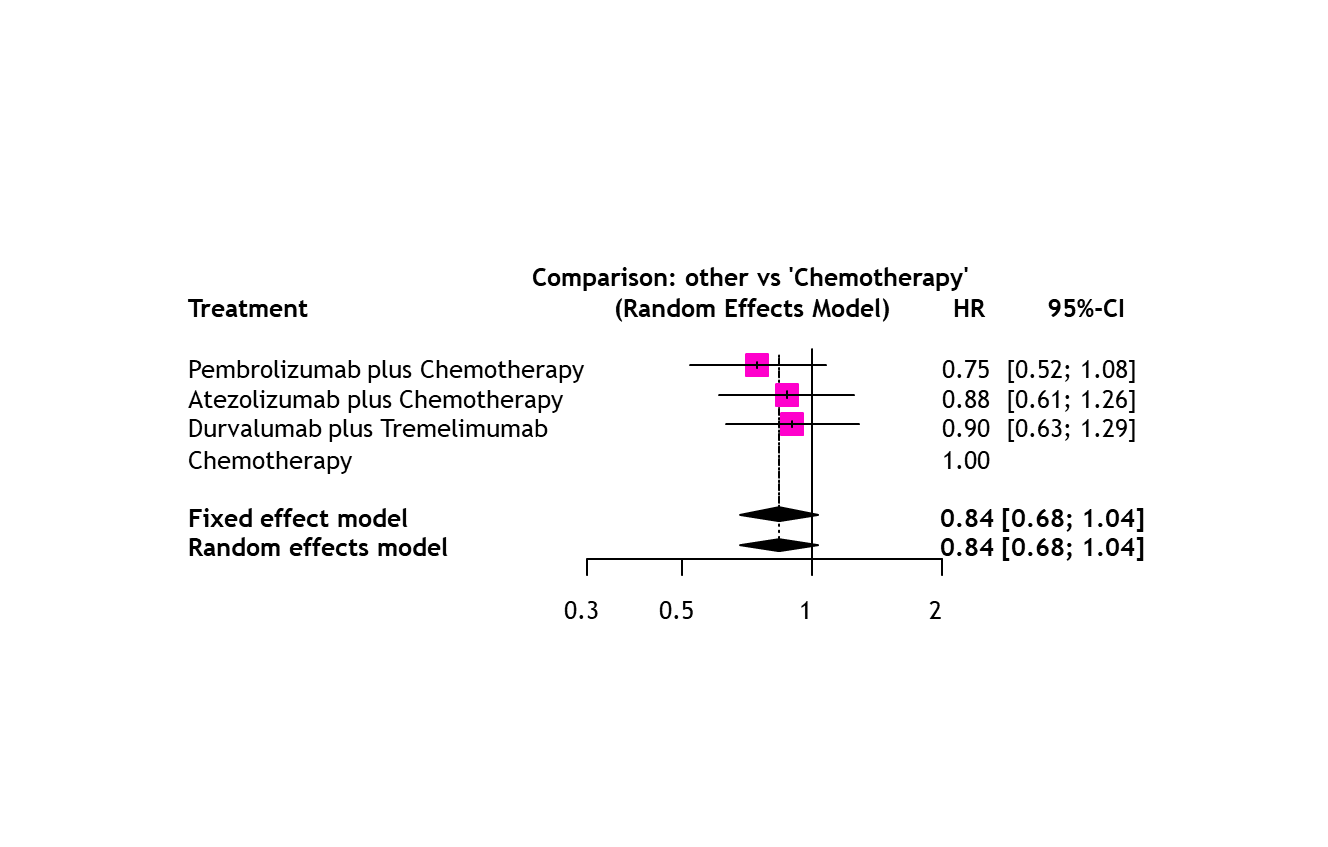

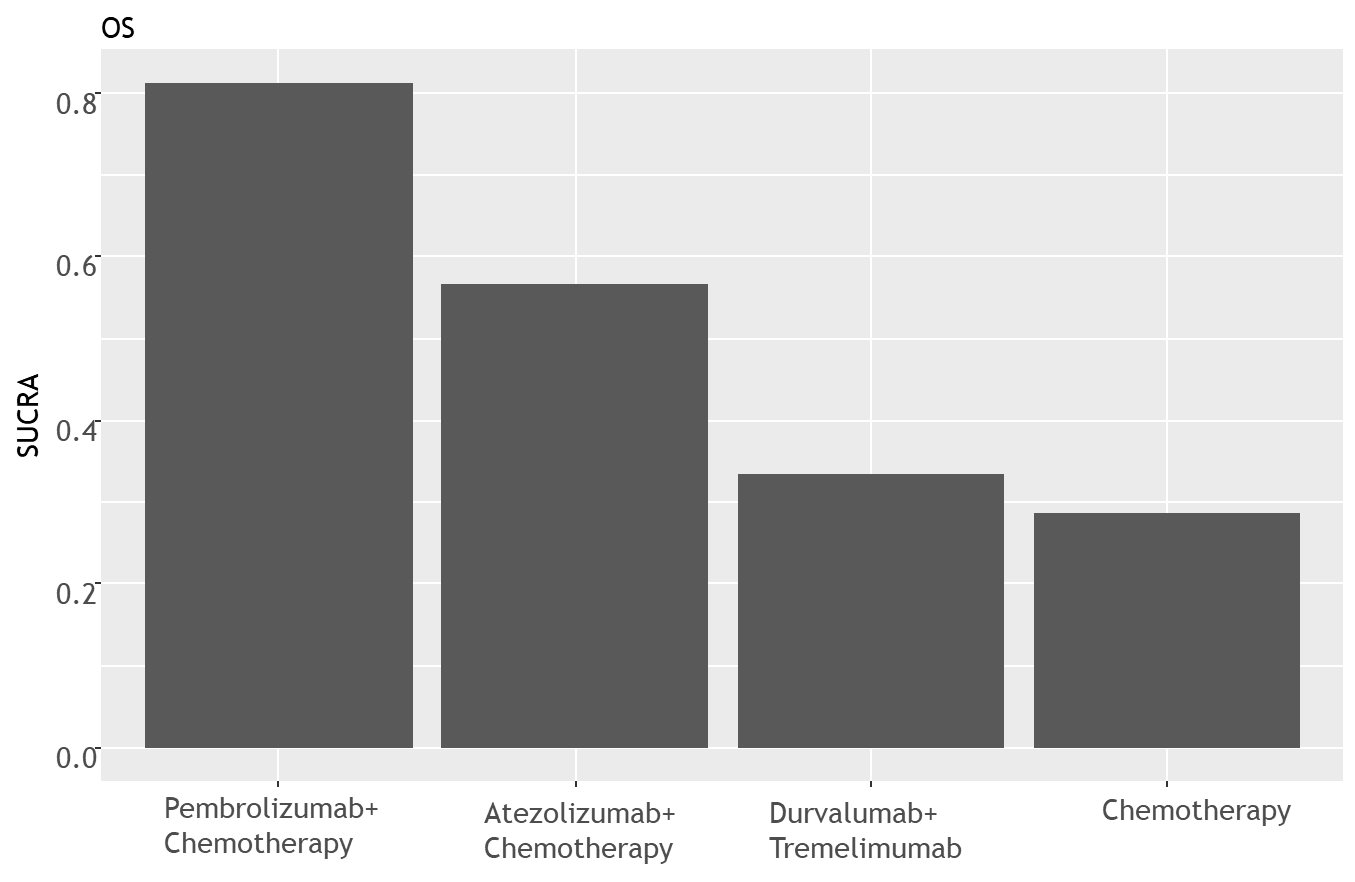
**
